# The Effects of Type 2 Diabetes Mellitus on Organ Metabolism and the Immune System

**DOI:** 10.3389/fimmu.2020.01582

**Published:** 2020-07-22

**Authors:** Gholamreza Daryabor, Mohamad Reza Atashzar, Dieter Kabelitz, Seppo Meri, Kurosh Kalantar

**Affiliations:** ^1^Autoimmune Diseases Research Center, Shiraz University of Medical Sciences, Shiraz, Iran; ^2^Department of Immunology, School of Medicine, Fasa University of Medical Sciences, Fasa, Iran; ^3^Institute of Immunology, University of Kiel, Kiel, Germany; ^4^Department of Bacteriology and Immunology and the Translational Immunology Research Program (TRIMM), The University of Helsinki and HUSLAB, Helsinki University Hospital, Helsinki, Finland; ^5^Department of Immunology, School of Medicine, Shiraz University of Medical Sciences, Shiraz, Iran

**Keywords:** immunometabolism, infectious diseases, insulin resistance, obesity, systemic low-level inflammation, SARS-CoV-2, type 2 diabetes mellitus

## Abstract

Metabolic abnormalities such as dyslipidemia, hyperinsulinemia, or insulin resistance and obesity play key roles in the induction and progression of type 2 diabetes mellitus (T2DM). The field of immunometabolism implies a bidirectional link between the immune system and metabolism, in which inflammation plays an essential role in the promotion of metabolic abnormalities (e.g., obesity and T2DM), and metabolic factors, in turn, regulate immune cell functions. Obesity as the main inducer of a systemic low-level inflammation is a main susceptibility factor for T2DM. Obesity-related immune cell infiltration, inflammation, and increased oxidative stress promote metabolic impairments in the insulin-sensitive tissues and finally, insulin resistance, organ failure, and premature aging occur. Hyperglycemia and the subsequent inflammation are the main causes of micro- and macroangiopathies in the circulatory system. They also promote the gut microbiota dysbiosis, increased intestinal permeability, and fatty liver disease. The impaired immune system together with metabolic imbalance also increases the susceptibility of patients to several pathogenic agents such as the severe acute respiratory syndrome coronavirus 2 (SARS-CoV-2). Thus, the need for a proper immunization protocol among such patients is granted. The focus of the current review is to explore metabolic and immunological abnormalities affecting several organs of T2DM patients and explain the mechanisms, whereby diabetic patients become more susceptible to infectious diseases.

## Introduction

The metabolic syndrome is defined by the presence of metabolic abnormalities such as obesity, dyslipidemia, insulin resistance, and subsequent hyperinsulinemia in an individual (1). Dyslipidemia, the main characteristic of metabolic syndrome, is defined by decreased serum levels of high-density lipoproteins (HDLs) but increased levels of cholesterol, free fatty acids (FFAs), triglycerides (TG), VLDL, small dense LDL (sdLDL), and oxidized LDL (ox-LDL) (Table 1) (2). Individuals with the metabolic syndrome are much more likely to develop type 2 diabetes mellitus (T2DM), cardiovascular diseases (CVDs), and fatty liver disease (2–4). T2DM, the most common form of diabetes (~90%), is characterized by a systemic inflammatory disease accompanied by insulin resistance (IR) or decreased metabolic response to insulin in several tissues, including the adipose tissue, liver, and skeletal muscle, as well as by reduced insulin synthesis by pancreatic beta cells (4, 5).

**Table 1:** Effects of type 2 diabetes mellitus on biochemical markers, as well as circulatory, digestive, and muscular systems.

		**Decreased or impaired**	**Increased**
Biochemical markers		HDL, lipid-binding capability of APO-A1, circulating H_2_S	Blood sugar, HbA1c, MGO, AGEs, ox-LDL, sdLDL, FFAs, TG, GrB, angiopoietin-1/2, EPO, VEGF-A, resistin, SCGN, homocysteine, elastase, proteinase-3, MPO, sFasL
Circulatory system	ECs	miR-Let7a, miR-26a, miR-126, mitochondrial membrane potential, catalase, superoxide dismutase, eNOS, NO	NF-κB1, caspase-3, Apoptosis, ROS, ICAM-1, IL-8, EMPs
	CAPCs cells	VEGFR-1 expression	VEGFR-2 expression, Apoptosis
	Platelets	miR-126 expression	Activity, prothrombotic state, MPV, MPs generation, sP-selectin and sCD40L induction, p2y12 receptor expression
Digestive system	IECs	GSH levels	Permeability, DMT1 expression, intestinal iron uptake, iNOS, NO
	Pancreatic beta-cells	PDX-1 expression, insulin synthesis	Conversion into α- and δ-“like” cells, ER stress, caspase-3 expression, Apoptosis, ROS generation, mitochondrial dysfunction, proteasomal dysfunction, insoluble IAPP induction
	Liver	miR-206	Steatosis, NF-κB1, STAT3
Muscular system	Skeletal muscle cells	GLUT-4 expression	NF-κB1, TNF-α, IL-6, IL-8, IL-15, MCP-1, GRO-α, and follistatin expression

*AGE, advanced glycation end product; APO, apolipoprotein; CAPCs, circulating angiogenic progenitor cells; CD, cluster of differentiation; DMT1, divalent metal transporter 1; ECs, endothelial cells; EMPs, endothelial microparticles; eNOS, endothelial nitric oxide synthase; EPO, erythropoietin; ER, endoplasmic reticulum; FFA, free fatty acid; GLUT-4, glucose transporter type 4; GrB, granzyme B; GRO, growth-regulated oncogene; GSH, glutathione; H2S, Hydrogen Sulfide; Hb, hemoglobin; HDL, high-density lipoprotein; IAPP, islet amyloid polypeptide; ICAM, intercellular adhesion molecule; IECs, intestinal epithelial cells; IL, interleukin; iNOS, inducible nitric oxide synthase; LDL, low-density lipoprotein; MCP-1, monocyte chemoattractant protein-1; MGO, methylglyoxal; miR, micro RNA; MP, Microparticle; MPO, Myeloperoxidase; MPV, mean platelet volume; NF-κB1, nuclear factor kappa-light-chain-enhancer of activated B cells 1; NO, nitric oxide; ox-LDL, oxidized LDL; PDX-1, pancreatic and duodenal homeobox 1; ROS, reactive oxygen species; SCGN, secretagogin; sdLDL, small dense LDL; sFasL, soluble Fas ligand; TG, triglyceride; TNF, Tumor necrosis factor; VEGF, vascular endothelial growth factor; VEGFR, VEGF receptor*.

**Figure 1:**
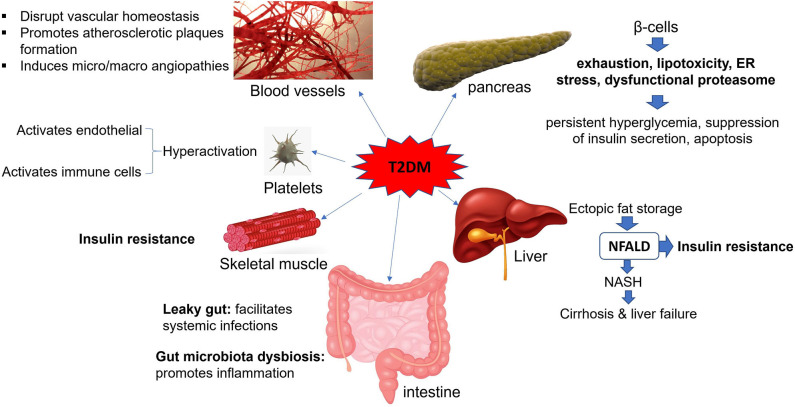
Effects of T2DM on body organs. T2DM is an inflammatory state that affects circulatory system, gastrointestinal tract, pancreatic beta cells, liver, and skeletal muscles and makes them dysfunctional. NFALD, non-alcoholic fatty liver disease; NASH, non-alcoholic steatohepatitis; ER, endoplasmic reticulum.

Studies on immunometabolism have indicated that the metabolic states and immunological processes are inherently interconnected (6). In this scenario, metabolites derived from the host or microbiota regulate immunological responses during health and disease (6). Accordingly, in obese individuals, expanded adipose tissue at different locations, by initiating and perpetuating the inflammation, induces a chronic low-level inflammatory state that promotes IR (4). Every organ system in human body can be affected by diabetes, but the extent of organ involvement depends largely on the severity and duration of the disease (Figure 1 and Table 1). During the progression of diabetes, hyperglycemia promotes mitochondrial dysfunction and induces the formation of reactive oxygen species (ROS) that cause oxidative stress in several tissues such as blood vessels and pancreatic beta cells (7–9). Accumulating damage to the mitochondria, as well as several macromolecules, including proteins, lipids, and nucleic acids by ROS promotes the process of aging (10). As a result, pancreatic β cells that require functional mitochondria to maintain insulin synthesis fail to generate high enough levels of insulin (11, 12). In the absence of compensatory mechanisms, stress-responsive intracellular signaling molecules are activated and cellular damage occurs. Elevated intracellular levels of ROS and subsequent oxidative stress play an important role in the pro-atherosclerotic consequences of diabetes and the development vascular complications (9, 13). Moreover, the non-enzymatic covalent attachment of glucose and its toxic derivatives [e.g., glyoxal, methylglyoxal (MGO), and 3-deoxyglucosone] to the biological macromolecules such as nucleic acids, lipids, and proteins leads to the formation of advanced glycation end products (AGEs) (14, 15). Accumulated AGEs block the insulin signaling pathway and promote inflammation (16, 17). In addition, the attachment of AGEs to their receptors [e.g., CD36, galectin-3, scavenger receptors types I (SR-A1), and II (SR-A2)] on the surfaces of immune cells in the circulation and tissues activates the expression of pro-inflammatory cytokines and increases free radical generation (18). Furthermore, due to the chronic exposure of cells to high glucose levels in untreated T2DM patients, glucose toxicity might occur in several organs. This will eventually lead to nephropathy, cardiomyopathy, neuropathy, and retinopathy.

Gut microbiome dysbiosis is another important factor that can facilitate the induction and progression of metabolic diseases such as T2DM (19). The gut microbiome dysbiosis, by altering the barrier functions of intestine and the host metabolic status, promotes the insulin resistance in diabetic patients (19). Diabetes also impairs the immune system and increases the susceptibility of patients to serious and prolonged infections (20). This is likely to be the case with the severe acute respiratory syndrome coronavirus 2 (SARS-CoV-2), as well (21, 22). In the current paper we will review recent research to explore the impairment of body organs in T2DM patients and explain how diabetic patients become more susceptible to certain infectious diseases.

## Effects of T2DM on the Circulatory System

**Figure 2:**
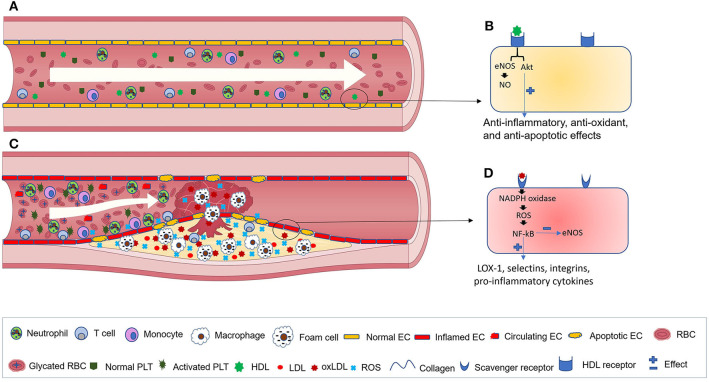
Blood vessels in healthy individuals and T2DM patients. **(A)** normal blood flow in healthy individuals. **(B)** A close view of HDL binding to its receptors on the surface of ECs that results in the activation of anti-inflammatory cascades. **(C)** Blood vessels in T2DM patients. During the progression of the disease, red blood cells become glycated, while activated ECs synthesize elevated levels of adhesion molecules and chemokines that facilitate monocytes recruitment, adhesion, and transmigration across the endothelium toward the subendothelial region. Monocytes are then differentiated into macrophages and eventually, by excess lipid uptake, generate foam cells. Subsequently, further immune cell infiltration into the atherosclerotic lesion occurs, where their inflammatory cytokines promote platelet activation, EC apoptosis, and increased generation of ROS and Ox-LDL. **(D)** interactions between oxLDL and its receptor aggravate ROS generation, NF-κB activation and inflammation. EC, endothelial cell; RBC, red blood cell; PLT, platelet; HDL, high-density lipoprotein; Ox-LDL, Oxidized low-density lipoprotein; ROS, reactive oxygen species; eNOS, endothelial nitric oxide synthase; NO, Nitric oxide; LOX-1, lectin-type oxidized LDL receptor 1.

Vascular homeostasis is an important function of the endothelium. Under homeostatic conditions, the ECs maintain the integrity of blood vessels, modulate blood flow, deliver nutrients to the underlying tissues, regulate fibrinolysis and coagulation, control platelet adherence and patrol the trafficking of leukocytes (Figure 2A) (23). Normal ECs also internalize high-density lipoproteins (HDLs) and its main protein part apolipoprotein A-I (apoA-I) in a receptor-mediated manner to activate endothelial cell nitric oxide (eNOS) synthase and promote anti-inflammatory and antiapoptotic mechanisms (Figure 2B) (24). HDL receptors on the surfaces of ECs include: the ATP-binding cassette (ABC) transporters A1 and G1, the scavenger receptor (SR)-B1 and the ecto-F1-ATPase (24).

According to the epidemiological studies, diabetes mellitus is considered as one of the main risk factors for CVD (Figure 1) (25). From the beginning of T2DM, the functions of ECs are impaired, which is the main cause of disease-related side-effects (26). ECs can initiate and perpetuate the inflammatory milieu during the pathogenesis of diabetes. Due to the negative impacts of hyperglycemia and subsequent oxidative stress, CVDs are more common among diabetic patients (27). It has been observed that incubation of human aortal endothelial cells (HAECs) with a medium containing high glucose concentrations (HG, 20 mM) increases the intracellular levels of MGO and glycated proteins that in turn activate the unfolded protein response (UPR) and trigger inflammatory and prothrombotic pathways (28). Glycated apoA-I, which is formed during hyperglycemia, modifies its structure, decreases its lipid-binding ability, prevents cholesterol efflux from macrophages and impairs its anti-inflammatory function (29, 30). Vaisar et al. have shown that HDLs from diabetic patients have a reduced capacity to trigger eNOS production and suppress tumor necrosis factor-α (TNF-α)-mediated inflammatory responses within ECs (31).

Diseases such as T2DM that induce high levels of vascular injury are accompanied by an elevated number of circulating endothelial cells (CECs) (32). T2DM-related risk factors such as dyslipidemia, hyperglycemia, and hyperinsulinemia as well as other conditions (e.g., inadequate physical activity, smoking, and high blood pressure) facilitate the formation of atherosclerotic plaques/lesions (33). Dyslipidemia, due to the elevated flux of FFA from insulin-resistant tissues and spillover from entry into adipocytes, is considered as an important risk factor for developing CVD among diabetic patients. This is because dyslipidemia promotes inflammation, endothelial dysfunction, and platelet hyperactivation (34, 35). During the progression of atherosclerosis, lipids, immune cells, and extracellular matrix accumulate in the arterial intima or subendothelial regions (Figure 2C) (33). Advanced plaques can impede blood flow and cause tissue ischemia or might become disrupted and generate a thrombus that stops the blood flow of important organs. Vascular complications of diabetes engage either tiny or large blood vessels (micro- and macroangiopathy, respectively). Microangiopathies, which can be seen in the kidneys, vasa nervorum and eye tissues, cause nephropathy, neuropathy, and retinopathy.

Macroangiopathies, by inducing atherosclerosis in the coronary, carotid, and peripheral arteries, increase the risk of myocardial infarction (MI), stroke and peripheral artery disease (PAD). Macrovascular complications due to EC dysfunction are considered as an important cause of mortality and morbidity among diabetic patients (36). Oxidative stress has an essential role in the induction of vascular complications during the course of diabetes (8). EC dysfunction (e.g., delayed replication, dysregulated cell cycling, and apoptosis), as well as enhanced ox-LDL formation are some consequences of oxidative stress. It has been well-established that sdLDL and ox-LDL have an enhanced atherogenic ability and are more useful biomarkers than total LDL for predicting CVD (37, 38). sdLDL particles have a smaller size than other LDL particles. Thus, sdLDL particles are more easily oxidized, and their atherogenic potential is enhanced. During oxidative stress, levels of ox-LDL increase by the excess action of reactive oxygen species (ROS) (13). Subsequently, ox-LDL interaction with scavenger receptors, including CD36, SR-A1/CD204, SR-B1, and lectin-like ox-LDL receptor-1 (LOX-1) on the surface of ECs activates the NADPH oxidase that in turn increases the expression of ROS and activates the transcription factor NF-αB (39). Afterwards, the expression of LOX-1, adhesion molecules (e.g., selectins and integrins) and the secretion of pro-inflammatory cytokines and chemokines are increased, while NO synthesis is decreased in ECs (Figure 2D) (39–41). EC-derived chemokines bind to their cognate receptors on the surfaces of monocytes and recruit them toward the inflamed endothelium. Following this, selectin-based rolling and integrin-based attachment of monocytes to the ECs cause their migration toward the subendothelial region, where they develop into lipid-laden macrophages or foam cells later on (42).

The scavenger receptor LOX-1 plays an important role in the uptake of ox-LDL during atherogenesis. It is strongly expressed on the surfaces of ECs, but has an inducible pattern of expression on the surface of macrophages and smooth muscle cells (43). The accelerated uptake of ox-LDL by macrophages accounts for their transformation into foam cells, the initial hallmark of atherosclerosis (41, 43). Besides, diabetes leads to both quantitative and qualitative defects in circulating angiogenic progenitor cells (CAPCs) that take part in the repair of injured endothelium (44). It has been shown that humans or mice with decreased numbers of CD31^+^CD34^+^CD133^+^CD45^dim^Sca-1^+^Flk-1^+^ CAPCs have an increased prevalence of T2DM, elevated HbA1c levels and aggravated CVD risk scores (44, 45).

In diabetic patients, despite elevated serum levels of pro-angiogenic molecules, like angiopoietin-1/2, EPO, and VEGF-A, angiogenesis is impaired. This is mainly due to the decreased expression levels of VEGFR2 and CXCR4 on the surfaces of CAPCs, which makes them unresponsive to the angiogenic factors (44, 46). It has also been shown that circulating proangiogenic granulocytes composed of eosinophils and neutrophils are also impaired in diabetic patients (47). Besides, elevated levels of AGEs in T2DM cause EC dysfunction and vascular inflammation (48). Ren et al. have shown that incubation of human coronary artery endothelial cells (HCAECs) with AGEs causes decreased expression (at both mRNA and protein levels) and enzymatic activity of eNOS, increased levels of ROS, diminished mitochondrial membrane potential and declined activity of catalase and superoxide dismutase in treated cells (49). Another study by Lan et al. has shown that AGEs in the pancreas decrease EC viability and induce their apoptosis in an NFκB signaling-related manner (50). However, apigenin (4′,5,7-trihydroxyflavone) can protect ECs against oxidative stress and subsequent inflammatory reactions mediated by AGEs (51). Apigenin binds to methylglyoxal (MGO) and forms a complex that inhibits AGE formation.

Chettab et al. have shown that the expression of ICAM-1 as well as the production of IL-8, are significantly increased in HUVECs cultured in HG medium compared to cells cultured in normal glucose (NG, 5.5 mM) conditions (52). Bammert et al. found out that incubation of HUVECs with HG media promotes the generation of endothelial microparticles (EMPs) that, when added to normally cultured HUVECs, downregulate the expression of anti-apoptotic microRNA miR-Let7a, but enhance the synthesis of active caspase-3 and cause cell apoptosis (53). Several microRNAs, including miR-21, miR-26a, miR-30, miR-92a, miR-126, miR- 139, miR-199a, miR-222, and miR-let7d, regulate vascular homeostasis. It has been shown that the expressions of miR-26a and miR-126 are significantly reduced in circulating MPs isolated from diabetic patients compared with normal individuals. This could be involved in making diabetic individuals more susceptible to coronary heart disease (54). Moreover, HG media upregulate the expression of NADPH oxidase that will induce the generation of ROS. This leads to subsequent apoptosis of the HUVECs through a ROS-dependent caspase-3 pathway (55).

Su et al. have demonstrated that argirein medication, by inactivating NADPH oxidase, can prevent endothelial cell apoptosis in a rat model of T2DM and hence attenuate vascular dysfunction (56). HG further increases the permeability of the HUVECs in a protein kinase C (PKC)-dependent manner (57, 58). Hassanpour et al. showed that incubation of endothelial progenitor cells with the serum of T2DM patients inhibits their migration toward bFGF, increases their expression of VEGFR-2, but reduces their expression of VEGFR-1 and induces their apoptosis (59). However, humanin (HN), a mitochondrium-derived peptide, is cytoprotective against apoptosis during pathological conditions, such as diabetes mellitus (60). It has been demonstrated that simultaneous incubation of H9C2 cells, a line of rat cardiac myoblasts, with H_2_O_2_ and HN decreases the intracellular levels of ROS, preserve mitochondrial function/structure and decline cellular apoptosis (61). Wang et al. have indicated that the treatment of HUVECs with HN before their incubation with HG medium increases the expression of eNOS, while decreasing the expression of endothelin 1 (ET-1), VCAM-1, TNF-α, IL-1β, and E-selectin in a krüppel-like factor 2 (KLF2)-dependent manner. Such changes in the expression of integrins prevent the attachment of monocytes to HUVECs (62). Accordingly, HN might be used to prevent the development of hyperglycemia-associated EC dysfunction in T2DM.

EC activation and expression of adhesion molecules also facilitate activation and adhesion of platelets. This will increase the risk of thrombosis and promote the development of thrombotic angiopathy, typical for diabetic patients. Platelets are tiny anucleated cellular fragments generated from megakaryocytes in the bone marrow. They circulate in the blood for ~5–9 days and play essential roles in hemostasis and in controlling vascular integrity (63). Circulating inactive platelets move in the proximity of vessel walls (Figure 2A) and rapidly get activated in response to vascular injury. At the end of their life, platelets are cleared from circulation with the action of the liver and spleen-resident macrophages. Platelets have an essential role in the initiation and progression of inflammation. Platelet hyperactivation that occurs during inflammatory states (e.g., T2DM) facilitates the pathogenesis of CVDs (Figure 2C) (64, 65). It has been shown that elevated levels of resistin, an adipokine, in diabetic patients enhances oxidative stress, promotes endothelial dysfunction and facilitates platelet activation (66). Activated platelets with an increased mean volume [mean platelet volume (MPV)] secrete microparticles (MPs) and soluble adhesion molecules (e.g., sP-selectin and sCD40L) that in turn activate endothelial and immune cells (67–69). Higher levels of platelet-derived MPs, which correlate positively with fasting blood sugar and glycated hemoglobin, have been shown in newly diagnosed T2DM patients compared to healthy individuals (70).

In T2DM patients thrombotic microangiopathies can lead to the development of CVDs (71). Platelets in the patients adhere to ECs and aggregate more rapidly than in healthy individuals thereby increasing the risk of thrombosis. In a mouse model of T2DM, Zhu et al. have shown that AGEs interact with CD36, a member of the type 2 scavenger receptor family, on the surfaces of murine platelets to activate them and induce a prothrombotic state (72). Elevated levels of the P2Y12 receptor on the surface of platelets in T2DM expose diabetic patients to a prothrombotic condition. This receptor has an essential role in platelet activation (73). Zhou et al. have shown that long non-coding RNA (lncRNA) metallothionein 1 pseudogene 3 (MT1P3), which is markedly upregulated in megakaryocytes of T2DM patients, enhances the expression of p2y12 receptor in platelets (74). They indicated that this is due to the inhibitory action of MT1P3 on miR-126.

## Effects of T2DM on the Digestive System

Virtually all parts of the human digestive system, including the gastrointestinal tract, pancreas, and the liver are affected by diabetes.

### Gastrointestinal Tract (GIT)

The GIT is populated with a myriad of microorganisms, including principally bacteria but also archaea, viruses, fungi, and protozoans that dynamically influence the health status and homeostasis of the host. The physiological functions of the GIT resident microbes improve gut integrity, protect against microbial pathogens and regulate immune responses (75). Mucosal barriers, such as intestinal epithelial cells (IECs) and the mucus layer, spatially isolate the host immune system and gut microbiota to prevent unnecessary immune activation and intestinal inflammation. They also facilitate the uptake of nutrients through receptors and transporters. However, hyperglycemia, in a GLUT2-dependent manner, can influence the mucus and alter the integrity of adherence and tight junctions between intestinal epithelial cells of diabetic mice. This will enhance the permeability of the intestinal barrier leading to so called “leaky gut.” Subsequently, hyperglycemia may facilitate the dispersal of an enteric infection into a systemic infection (Figure 1) (76). Interestingly, the reversal of hyperglycemia, conditional deletion of GLUT2 from the IECs and inhibition of glucose metabolism will fix the barrier dysfunction and prevent the spread of bacteria (76). Xu et al. have shown that *Faecalibacterium prausnitzii*, one of the most frequent commensal bacteria in normal individuals with essential roles in gut homeostasis, generates anti-inflammatory molecules that enhance the expression of tight junctions and improve intestinal integrity during diabetes (77). However, in some cases, gut microbiota dysbiosis or altered microbial composition of the intestines could induce T2DM and lead to its progression (78).

Of interest, the widely used antidiabetic drug metformin can improve barrier integrity and restore the healthy microbiota composition of the gut in diabetic patients (79). The intestinal commensal bacterium *Akkermansia muciniphila* can also act as a sentinel to reduce microbial translocation across the gut and prevent the subsequent inflammation in patients with T2DM (80). Hyperglycemia can further decrease the intracellular levels of glutathione (GSH) but increase iNOS activity and NO production in the IECs (81). Zhao et al. have found out that hyperglycemia in a PKCα-dependent manner inhibits the ubiquitination, internalization and degradation of the divalent metal transporter 1 (DMT1) present on the microvillar membranes of IECs. Subsequently, intestinal iron uptake is enhanced and accumulated iron ions aggravate diabetes-related complications and increase mortality (82, 83).

### Pancreas

The pancreas consists of the exocrine and endocrine compartments. The endocrine part is made of different cell types, including α, β, δ, and ε cells that secrete glucagon, insulin, somatostatin, and ghrelin hormones, respectively. These cells are aggregated into specialized structures called islets of Langerhans, which play an important role in controlling blood glucose levels through the secretion of insulin and glucagon. In T2DM, despite normal levels of β-cell replication and islet formation, β-cell apoptosis is increased so that the number of cells declines by ~50% (Figure 1) (84). During the progression of T2DM, the insulin-resistant state forces β-cells to compensate for the lack of insulin by elevating its synthesis to restore the normal blood glucose level. However, in severe diabetic patients, β-cell exhaustion, and subsequent persistent hyperglycemia occur (7). Furthermore, chronic elevated serum levels of free fatty acids, seen in obesity and T2DM, induce lipotoxicity in beta-cells and suppress their insulin secretion ability (85). To alleviate chronic inflammation, overcome insulin resistance (IR) and to prevent β-cell apoptosis, stem cells or stem cell derivatives such as insulin-producing cells (IPCs) and exosomes have been suggested (86–89). Their effects are believed to be mainly due to their anti-inflammatory activities.

Secretagogin (SCGN) is predominantly expressed by pancreatic β-cells protecting their normal functions. SCGN also acts as an insulin binding protein to make it more stable, avoid its aggregation, improve its functions and enhance its secretion (90, 91). In T2DM patients, due to the islet cell dysfunction and endoplasmic reticulum (ER) stress, serum levels of SCGN are elevated reflecting stress and dysfunctional islet cells (92). Moreover, in patients with T2DM, islet amyloid polypeptide (IAPP or amylin), a peptide hormone and one of the main secretory products of pancreatic β-cells, tends to deposit in the islets of Langerhans, form insoluble fibrils and impair secretory functions of β-cells (93). IAPP is costored with insulin in the secretory granules of pancreatic β cells. In steady-state conditions it regulates food intake, insulin secretion, and glucose metabolism (94). Ribeiro et al. have noted that pancreatic extracellular vesicles (EVs) from healthy individuals, but not from T2DM patients, directly bind to IAPPs and prevent amyloid formation within the pancreatic islets (95). The authors showed that the altered protein-lipid composition of the EVs is the main reason for this discrepancy (95). However, Chatterjee et al. have shown that β-cells from T2DM patients have a dysfunctional proteasome complex that fails to degrade pancreatic IAPP, whereby amyloid formation is induced (96). Furthermore, in T2DM patients, lipids accelerate the formation of fibrillary IAPP, which aggravates islet cell damage (97).

Dhar et al. have demonstrated that chronic use of MGO in Sprague-Dawley rats increases the expression of NF-αB, MGO-derived AGEs and their receptors in pancreatic β cells. MGO can also induce apoptosis of islet β cells, increase fasting plasma glucose levels and impair glucose tolerance (98). In T2DM patients the plasma level of MGO directly correlates with fasting blood sugar and HbA1c levels (99). Bo et al. further showed that MGO in a dose-based manner impairs insulin secretion of pancreatic β-cell lines MIN6 and INS-1 through increased generation of ROS and by induction of mitochondrial dysfunction (100). Robertson et al. have found out that elevated levels of ROS in pancreatic β-cells inhibit the pancreas duodenum homeobox-1 (PDX-1) transcription factor that is needed for insulin synthesis (7). It has been shown that chronic use of MGO in animals could induce T2DM, while simultaneous use of alagebrium, which breaks AGE compounds, attenuates the disease (98). It has also been reported that during the course of diabetes dedifferentiation and conversion of β-cells into α- and δ-“like” cells occurs (101). In conclusion, the pancreatic β cell function is progressively reduced during the progression of T2DM.

### Liver

The liver is by far the most important metabolic organ with essential roles in regulating homeostasis and mediating glucose and lipid metabolism. Metabolic activities of the tissue are precisely controlled by the actions of metabolic substrates, including free fatty acids (FFAs) and hormones (102). T2DM patients usually suffer from a chronic liver condition called non-alcoholic fatty liver disease (NAFLD). It is characterized by steatosis that means ectopic fat storage in hepatocytes and subsequent insulin resistance (Figure 1) (103). Lipid accumulation in hepatocytes leads to impaired biogenesis of miR-206 that facilitates insulin signaling and prevents lipogenesis (104). Several factors such as obesity, increased serum levels of fatty acids, and insulin resistance can increase the risk of fatty liver disease. P2Y2 receptor, through the induction of the c-Jun N-terminal kinase (JNK) and prevention of insulin signaling, can promote insulin resistance in hepatocytes in T2DM (105). In some cases, NAFLD may progress into an aggressive form of inflammatory fatty liver disease called non-alcoholic steatohepatitis (NASH), which might cause liver cirrhosis and organ failure (106). Dang et al. have indicated that exosomes released from the adipose tissues of obese mice due to the smaller miR-141-3p content can promote insulin resistance in the murine hepatocyte cell line AML12 (alpha mouse liver 12) (107). The adipokine visfatin that is released from the adipose tissue of obese individuals has also been shown to activate the pro-inflammatory STAT3 signaling pathway and NF-κB in the human liver cell line HepG2 and promote their insulin resistant state (108). Nevertheless, the hepatocyte growth factor (HGF) can alleviate the insulin resistance of hepatocytes and control their triglyceride and cholesterol contents (109).

## Effects of T2DM on Skeletal Muscles

Skeletal muscle (SM) is the main tissue that releases glucose after insulin stimulation. Hence, insulin resistance in SM has a pivotal role in the metabolic dysregulation of T2DM. Insulin resistance in SM is the primary defect of T2DM that facilitates the progression of fatty liver disease, deposition of fat in the liver (Figure 1) (110). Skeletal muscle from diabetic patients expresses less genes related to insulin signaling and metabolic pathways, but more apoptosis and immune-related genes (111). This inflammatory milieu is mainly due to the proinflammatory actions of obesity-related adipose tissue mediators, which are released into the circulation and promote inflammation within the SM (4). Furthermore, obesity causes intermyocellular and perimuscular adipose tissue expansion that acts like adipose tissue depots to enhance SM inflammation (112). It has been shown that human skeletal muscle cells (hSMC), isolated from diabetic patients, after a 24-h culture generate significantly more TNF-α, IL-6, IL-8, IL-15, monocyte chemotactic protein (MCP)-1, Growth-Related Oncogene (GRO)-α, and follistatin compared to non-diabetic individuals (113). This altered secretion of myokines (e.g., cytokines secreted by SMs) is an intrinsic feature of SM during the progression of T2DM. In SM, GLUT-4, which is quickly translocated to the cell surface, facilitates glucose uptake in response to insulin hormone as well as muscle contraction. Pinto-Junior et al. have shown that the use of AGE-albumin in rats increases the expression of the inflammatory molecule NF-κB1 within the SM. NF-κB1 binds to the promoter of the GLUT-4 gene and suppresses its expression (at both mRNA and protein levels) (114). Accordingly, GLUT-4 levels on the surfaces of SM decrease and subsequently, whole-body IR develops.

## Effects of T2DM on the Immune System

The immune system is generally classified into two main arms, innate and adaptive (or acquired) immunity. Adaptive immunity is mediated by B cells, which produce antibodies and T cells, which are classified into CD4^+^ helper cells and cytotoxic CD8^+^ cells. A considerable literature has discussed the dysfunctional immune responses in diabetic patients (Table 2) (115–120). Abnormal immune cell activation and subsequent inflammatory environment has an essential role in the progression of T2DM (121). In this regard, chronic inflammation due mainly to the activation of the myeloid cell lineage (e.g., macrophages and neutrophils), is directly related to the induction of IR (4, 122). Fang et al. have shown that patients with T2DM have elevated numbers of circulating leukocytes that express high levels of inflammatory gene products but glycemic control can reverse the situation (123). De Souza Prestes et al. have indicated that exposure of leukocytes to MGO changes their morphology by making them larger and more granular, increases their ability to produce ROS and decreases their expression of antioxidant genes (124). They further demonstrated that treatment with MGO increases the expression of the pro-apoptotic gene BAD, while decreasing the expression of anti-apoptotic gene BCL-2, and hence promotes apoptosis of leukocytes (124).

**Table 2:** Effects of T2DM on the immune system.

Total leukocytes	Their numbers are elevated, are larger and more granular, express diminished levels of antioxidant genes but elevated levels of pro-apoptotic and pro-inflammatory genes.
**Innate immunity**	
Complement system	Attachment of C-type lectin proteins to mannose residues is decreased, lectin pathway is impaired, CD59 activity is reduced, MAC deposition in vascular walls is increased.
Dendritic cells (DCs)	Their numbers and activity are reduced.
Macrophages	Their cholesterol efflux is decreased, generate foam cells, have dysfunctional efferocytosis.
Neutrophils	Are activated, constitutively release NETs, produce high levels of MPO, ROS, and calprotectin (S100A8/A9), are more susceptible to apoptosis, their migration, phagocytosis and microbial killing are impaired.
NK cells	Their numbers are increased but are usually dysfunctional, express high levels of GLUT4 but decreased levels of NKG2D and NKp46, have reduced degranulation capacity, are more susceptible to apoptosis.
NKT cells	Their numbers are increased, produce high levels of IFN-γ, IL-4, and IL-17, express high levels of NKp30, NKG2D, and NKp44 but low levels of NKG2A and 158b.
Innate lymphoid cells (ILCs)	ILC1s are increased and produce high levels of IFN-γ.
**Adaptive immunity**	
Humoral immunity (B cells)	Germinal centers are reduced, Ab production and isotype switching is defective, Abs become glycated, Abs fail to activate complement.
Cellular immunity (T-Cells)	Pathogen-specific Th17 cells are decresed, Th1 cells are elevated, have decreased expression of perforin, GrB and CD107a.

*Ab, antibody; GLUT-4, glucose transporter type 4; GrB, granzyme B; IFN, interferon; IL, interleukin; MAC, membrane attack complex; MPO, Myeloperoxidase; NET, neutrophil extracellular traps; NKG2D, the natural killer group 2d; ROS, reactive oxygen species; Th, helper T cell*.

Hu et al. have shown that polyinosinic:polycytidylic acid (polyI:C), a Toll-like receptor 3 (TLR3) agonist, stimulated PBMCs in HG medium (24 mM glucose), while cells cultured in LG produced significantly lower levels of type I IFNs (125). Additionally, they indicated that under these conditions PBMCs express elevated levels of IFN-γ, IL-1β, IL-6, IL-10, granulocyte-macrophage colony-stimulating factor (GM-CSF), TNF-α, RANTES/CCL5 (Regulated on Activation, Normal T cell Expressed and Secreted), and macrophage inflammatory protein (MIP)-1α (125). Hu et al. have also shown that HG in T2DM patients decreases the formation, viability, differentiation and functions of osteoclasts, which are bone-resident innate immune cells (126). This may affect bone structure and delay bone healing. Defects in the innate, as well as adaptive immunity, are supposed to be the main cause of diabetic individuals' susceptibility to infections (127). Furthermore, some microorganisms, especially bacteria, in hyperglycemic conditions are better nourished and become more virulent, while also having a better milieu to cause infections.

### Innate Immunity

#### Complement System

The complement system is a first-line defense mechanism against invading microorganisms. It acts via different but interconnected classical, alternative, and lectin pathways (128). Ilyas et al. have shown that under high glucose conditions, the attachment of C-type lectin proteins to high-mannose containing glycoproteins is substantially decreased in a dose-dependent manner. These carbohydrate-binding proteins include mannose-binding lectin (MBL), surfactant protein D (SP-D), dendritic cell-specific intercellular adhesion molecule-3-grabbing non-integrin (DC-SIGN, CD209), and DC-SIGN-related (DC-SIGNR) protein (129). Reduced binding of MBL in the presence of high levels of sugar causes a significant reduction in the lectin pathway activity, but does not influence classical or alternative pathway activity (129). Nevertheless, Barkai et al. did not find significant differences in the function of classical or MBL pathways between T2DM and healthy individuals (130). However, significantly decreased activity of ficolin-3-mediated lectin and alternative pathways, as well as decreased levels of C4d and soluble complement C5b-9 (sC5b-9) were seen in diabetic patients with *Escherichia coli-*mediated urinary tract infections (130). This may be linked to a reduced ability of diabetics to protect themselves against bacterial infections.

The lipopolysaccharides of certain Gram-negative bacteria, like *Salmonella* serotype O6,7 as well as the cell walls of fungi, are rich in mannose. Possibly, because of this, in addition to additional provision of nutrients, an increased prevalence of fungal infections is seen in T2DM patients (131, 132). Patel et al. found a significantly higher prevalence of oral candida carriage in diabetic patients compared to healthy controls (131). They found that *Candida albicans* was the most commonly isolated species followed by *C. tropicalis*, but uncommon species such as *C. lusitaniae* and *C. lipolytica* were also isolated (131). Another study by Jhugroo et al. showed that *C. albicans* is the predominant yeast isolated from oral mucosal lesions of diabetic patients, followed by. *C. tropicalis* and *C. krusei* (132). Chikazawa et al. have shown that AGEs are recognized by C1q, which subsequently activates the classical complement pathway (133). Qin et al. have previously reported that AGEs can inactivate the complement regulatory protein CD59 (protectin) and hence increase the deposition of membrane attack complex (MAC) in tissues and vascular walls of diabetic patients (134). Recently, Bus et al. demonstrated classical complement pathway activation within the kidneys of T2DM patients with diabetic nephropathy (DN), as revealed by deposition of C1q, C4d, and C5b-9 in the glomeruli and arterioles (135).

#### Dendritic Cells (DCs)

Dendritic cells (DCs) are a heterogeneous population of specialized and professional antigen-presenting cells (APCs) that create a crucial link between the innate and adaptive immune responses (136, 137). Some studies have shown that the numbers of DCs are reduced in both type 1 and 2 diabetes (138, 139). Seifarth et al. have found that T2DM patients with poor metabolic control have decreased numbers of both myeloid and plasmacytoid DCs compared with healthy controls. This could make them more susceptible to opportunistic infections (139). In the case of good blood glucose control, the reduction in DC numbers was less prominent but still significant, especially for myeloid DC1 (mDC1) cells (139). Another study by Blank et al. demonstrated that women with T2DM and poor glycemic control (HbA1c ≥ 7%) have fewer numbers of circulating plasmacytoid DCs (pDCs) compared to diabetic women with good glycemic control (HbA1c < 7%) or to healthy women (140). Montani et al. have recently shown that hyperglycemic medium and hyperglycemic sera derived from T2DM patients prevent the maturation of monocytes into effective DCs and their activation *in vitro* (141). Interestingly, quercetin, a flavonoid with anti-inflammatory and antioxidant characteristics, prevented such effects (141).

#### Macrophages

Macrophages are important immune cells that play critical roles through all stages of the pathogenesis of T2DM-related atherosclerosis (41). Swirski et al. have shown a significantly elevated number of pro-inflammatory monocytes in the circulation of ApoE^−/−^ mice, an animal model of atherosclerosis, compared to control mice (142). Modifications of the lipoproteins in the arterial walls of diabetic individuals make them pro-inflammatory and activate the overlying endothelium. In response, monocytes are recruited into the subendothelial region, differentiate into macrophages and internalize the accumulated lipoproteins. Finally, cholesterol-laden foam cells are generated. They promote inflammation and progression of the disease through the synthesis and secretion of cytokines, chemokines, ROS, and matrix metalloproteinases (MMPs) (Figure 2C) (42). Foam cells lose their migratory potential, die by apoptosis and generate a necrotic core within the atherosclerotic plaque (143).

It has been demonstrated that the use of mesenchymal stem cells in ApoE^−/−^ mice reduces the numbers of monocytes/macrophages at the site of inflammation, decreases lipid deposition and diminishes plaque size (144). Ma et al. have studied the effects of long-term hyperglycemia in diabetic mice and found out that compared to non-diabetic control mice, the numbers of F4/80^+^ macrophages isolated from spleen (SPMs), as well as from peritoneal exudates (PEMs) of diabetic mice are significantly decreased (145). Subsequently, Sun et al. showed that stimulation of PEMs from diabetic mice *in vitro* with IFN-γ and lipopolysaccharide (LPS) significantly decreased the expression of intercellular adhesion molecule 1 (ICAM-1 or CD54), CD86, TNF-α, and IL-6, while it increased the production of nitric oxide (NO) (146). They further showed that stimulation of PEMs isolated from diabetic mice with IL-4 caused an enhanced arginase activity (146). Kousathana et al. have demonstrated that circulating monocytes isolated from diabetic patients produce higher levels IL-6, while having an impaired activation of the NLRP3 inflammasome and subsequently reduced IL-1β production (147). However, they showed that proper glycemic control would restore such modifications. Poor inflammatory responses in circulating monocytes, as well as in macrophages, are responsible for elevated susceptibility to infections and their severity in patients with T2DM.

Macrophages play a critical role in tissue repair. Early in wound healing, they are pro-inflammatory to clear pathogens and debris but later, they resolve inflammation and promote tissue repair. In pathological conditions, failure to transform from pro-inflammatory to the anti-inflammatory proliferative phase can cause chronic inflammation in the affected tissue (148). Khanna et al. have indicated that dysfunctional phagocytosis of dead cells by macrophages (efferocytosis) at the wounds of diabetic mice expands apoptotic cell burden, causes chronic inflammation and prolongs wound healing (148). Mirza et al. have shown that an impaired wound healing process in animals with T2DM is due to high levels of NLRP3 inflammasome activity, which promotes the generation of IL-1β and IL-18 in macrophages (149, 150). Efficient skin wound healing process is mediated by the up-regulation of the peroxisome proliferator-activated receptor (PPAR)-γ in macrophages that convert their pro-inflammatory phenotype into healing-related. PPAR-γ suppresses cytokine production by macrophages and hence is upregulated in inflamed tissue-resident macrophages. However, in T2DM, PPARγ expression is down-regulated in skin-resident macrophages that enhance the activity of NLRP-3 inflammasome and cause chronic inflammation. Using myeloid-specific PPAR-γ^−/−^ mice, it has been shown that the absence of PPAR-γ in macrophages is sufficient to delay the healing process and extend tissue inflammation (150).

In T2DM patients, chronic hyperglycemia and hyperlipidemia trigger the secretion of a damage-associated S100A8 molecule (calgranulin A) from pancreatic islets that in turn increase macrophage infiltration (151). Westwell-Roper et al. have shown that IAPP aggregates in T2DM patients polarize islet-resident macrophages toward the M1-like F4/80^+^CD11b^+^CD11c^+^ phenotype that produces pro-inflammatory cytokines, including TNF-α, IL-1β, and IL-6. Furthermore, M1 cells promote islet inflammation, cause β-cell malfunction and apoptosis (152). In T2DM, excess phagocytosis of apoptotic β-cells by macrophages induces their lysosomal permeabilization, generation of ROS, inflammasome activation, and pro-inflammatory cytokines secretion (153). Collectively, these observations reveal that the functions and plasticity of macrophages are compromised during the progression of T2DM.

#### Neutrophils

Neutrophils are the most prevalent circulating leukocytes and one of the main components of innate immunity. They are recruited to the sites of infection through chemotaxis following complement activation, most importantly by C5a. Activated neutrophils bind via their surface receptors to induced ligands on the surfaces of inflamed endothelial cells to migrate to tissues. There they phagocytose and kill invading microbes with lysosomal enzymes, antimicrobial peptides and by the generation of ROS (154). Neutrophils from patients with T2DM, but not from healthy individuals, are activated and produce elevated levels of ROS. So, it could increase the risk of random organ injury (155). In diabetic patients, the plasma levels of homocysteine are elevated, which is mainly due to its impaired clearance rate (156). This will induce neutrophils to constitutively release neutrophil extracellular traps (NETs) that can cause vascular damage and delays in wound healing (157, 158). It has been shown that the circulating level of hydrogen sulfide (H_2_S) is significantly reduced in fasting blood of patients with T2DM compared with healthy individuals as well as in streptozotocin-induced diabetic rats compared with controls (159). H_2_S is produced from cysteine by the action of several enzymes. It acts as a regulator of cell signaling and homeostasis (160).

It is essential to maintain balanced levels of antioxidants and protect tissues from oxidative stress (160). The use of H_2_S or the endogenous L-cysteine *in vitro* blocks the production of IL-8 and monocyte chemoattractant protein-1 (MCP-1) in the human U937 monocyte cell line incubated in high-glucose medium (159). Yang et al. have shown that H_2_S treatment decreases NETosis and enhances the healing process of diabetic wounds by preventing ROS-dependent ERK1/2 and p38 activation (161). It has been shown that the levels of NET components, including histones, elastase and proteinase-3, are elevated in the sera from patients with diabetic foot ulcers (162). Wang et al. have recently indicated that HG dramatically enhances NADPH oxidase-dependent NET generation in diabetic rats and humans. It was proposed that this could have a role in the induction of diabetic retinopathy (163). Indeed, patients with T2DM have elevated plasma levels of MGO, which can induce the production of pro-inflammatory cytokines like TNF-α, IL-6, and IL-8 by neutrophils and make them more susceptible to apoptosis (99).

Myeloperoxidase (MPO), which is abundantly produced by neutrophils, but only to a small extent by monocytes and macrophages, might be useful as an early biomarker of inflammation in diabetic individuals (164). Binding of MPO to endothelial cells increases its half-life. Thereby, more pro-inflammatory oxidant hypochloric acid (HClO) is generated that extends the damage to blood vessels (165). In T2DM patients, neutrophil activities, including migration, phagocytosis and microbial killing are impaired. This makes diabetic individuals more susceptible to infections (166). It has been well-documented that neutrophils isolated in animal models of T2DM have an impaired TLR4 signaling pathway. This is reflected as a diminished cytokine and chemokine production, possibly as a consequence of reduced phosphorylation of NFκB and IκBα (167). The half-life of these neutrophils as well as their *in vivo* migration and myeloperoxidase activity are decreased.

During hyperglycemia, neutrophils produce calprotectin (S100A8/A9), which interacts with the receptor for advanced glycation end products (RAGE) on the surface of hepatic Kupffer cells and promotes the synthesis of IL-6 (168). Subsequently, IL-6 stimulates hepatocytes to increase the generation of thrombopoietin that in turn attaches to its receptor on the surfaces of bone marrow precursor cells and megakaryocytes to enhance their proliferation and expansion. This results in reticulated thrombocytosis, which means elevated megakaryocyte activity and thrombopoiesis. Interestingly, diabetes-related thrombocytosis and subsequent atherothrombosis can be reduced by lowering blood glucose, depleting Kupffer cells or neutrophils or by preventing the binding of S100A8/A9 to RAGE using paquinimod (168).

Thom et al. have shown that the incubation of human and murine neutrophils with HG medium would cause their cytoskeletal and membrane instability. This will induce the generation of 0.1 to 1 μm diameter microparticles and activate the NLRP3 inflammasome (169). Microparticles, which are potently pro-inflammatory, are found in the circulation of healthy individuals, but their generation is increased during cell activation in several diseases, including T2DM and cardiovascular diseases (170, 171). Furthermore, serum levels of soluble FasL (sFasL) are increased in patients with T2DM thereby activating neutrophils and aggravating the inflammatory milieu (172, 173). The proinflammatory roles of sFasL are mediated through increased amounts or activity of NFκB, IL-1β, caspase-1, CD11b/CD18, and ROS (173). Caspase-1 activation prevents the sFasL-dependent apoptosis of neutrophils and inhibits their expression of Fas and caspase-3 (173). Accordingly, hyperglycemia disturbs the normal functions of neutrophils and increases the susceptibility to infections by pathogenic microorganisms.

#### NK Cells

NK cells are innate lymphocytes that detect and directly kill virus-infected cells and tumor cells. They do not have similar specific receptors (TCR) for the recognition of distinct peptides as T cells do. Piatkiewicz et al. have observed that the numbers of NK cells in T2DM are increased, but most of them are dysfunctional. Diabetic NK cells express elevated levels of glucose transporter type 4 (GLUT4), which may render diabetic individuals more prone to colon cancer (174, 175). Berrou et al. showed that NK cells from T2DM patients express significantly decreased levels of activating receptors NKG2D and NKp46 and have a reduced degranulation capacity (176). Peraldi et al. indicated that the main cause of such changes is neutrophil-derived ROS (177). The expression level of NKG2D is negatively correlated with HbA1c levels implying that chronic hyperglycemia would cause NK cell dysfunction (176). Also, hyperglycemia increases the expression of unfolded protein response (UPR) genes in NK cells and induces their apoptosis (176).

#### NKT Cells

NKT cells express simultaneously markers of both T cells (TCR and CD3) and NK cells [CD16, CD56, CD314 (NKG2D), and CD337 (NKp30)]. NKT cell subsets produce a broad range of cytokines, including GM-CSF, IFN-γ, TNF-α, IL-2, IL-4, IL-5, IL-9, IL-10, IL-13, IL-17, and IL-21 (178). They recognize lipids and glycolipids presented by CD1d molecules. Phoksawat et al. have shown that the frequency of CD3^+^CD4^+^CD28^null^CD56^+^NKG2D^hi^ NKT cells, which produce high levels of IL-17, are increased in diabetic patients and their numbers are directly correlated with HbA1c levels (179, 180). Lv et al. have recently shown that the numbers of CD3^+^CD56^+^ NKT cells are higher in diabetic patients compared to healthy individuals (181). They further showed that such cells are mostly CD4^+^, produce elevated levels of IFN-γ and IL-4 and express high levels of NKp30, NKG2D, and NKp44 but low levels of inhibitory receptors NKG2A and 158b (181). The co-culture of these cells with HUVECs significantly decreased their proliferation and migration abilities that were mainly IL-4-dependent (181). Taken together these studies show that diabetic individuals appear to have elevated levels of inflammation-promoting NKT cells.

#### Innate Lymphoid Cells (ILCs)

ILCs are critical effectors of innate immunity that produce both regulatory and pro-inflammatory cytokines to promote tissue repair, immunity, and inflammation (182). Mature ILCs lack the TCRs. Based on their cell surface markers, cytokine production as well as expression of transcription factors the ILCs are classified into types 1, 2, and 3 (183). These correspond to the different types of CD4+ T helper cells: Th1, Th2, and Th17, respectively. IFN-γ is the cytokine signature of ILC1s, while type 2 cytokines (e.g., IL-5 and IL-13) are mainly produced by ILC2s and the main product of ILC3s are IL-17 and IL-22. Regarding transcription factors, T-bet is mainly expressed by ILC1s, GATA3 and RORα are mostly expressed by ILC2s and RORγt is predominantly expressed by ILC3 (183).

In T2DM, the numbers of circulating as well as adipose tissue-resident ILC1s are increased compared with normal individuals (184, 185). The frequency of circulating ILC1s is positively correlated with fasting plasma glucose (FPG), HbA1c, homeostasis model assessment for insulin resistance (HOMA-IR), serum-free fatty acids (FFAs) and adipose tissue insulin resistance index (Adipo-IR) (184, 185). It has also been shown that patients with increased numbers of ILC1 have an elevated risk of developing T2DM (184). A study by Wang et al. indicated that adipose tissue-resident ILC1s, via the production of IFN-γ, promote tissue fibrosis and induce diabetes in obese individuals (185). Liu et al. have demonstrated that the numbers of ILC2s as well as serum cytokine levels of IL-4, IL-5, and IL-13 are significantly elevated in diabetic kidney disease patients and have a positive correlation with disease severity (186). They further demonstrated that ILC2s, through the TGF-β1 signaling pathway, are involved in renal fibrosis seen in diabetic kidney disease (184). However, Galle-Treger et al. indicated that the engagement of the glucocorticoid-induced tumor necrosis factor receptor (GITR/or TNFRSF18) on the surface of activated ILC2s promotes their secretion of IL-5 and IL-13, ameliorates glucose homeostasis, protects against the onset of and improves established insulin resistance (187). The protective role of ILC2s during acute metabolic stress has also been well-documented by Dalmas et al. (188).

### Impairment of Adaptive Immunity in T2DM

#### Humoral Immunity (B Cells)

Elevated levels of blood glucose generate covalent sugar adducts with several proteins through non-enzymatic glycation. This can impair humoral immunity in many ways, e.g., by modifying the structure and functions of immunoglobulins (Igs) (189–194). Such modifications in the structure of Igs can be determined using matrix-assisted laser desorption ionization (MALDI) mass spectrometry (119, 191). The molecular mass of Igs in diabetic patients is higher than in normal subjects (189). This can lead to reduced efficiency of vaccines that stimulate humoral immunity in these patients. It has been shown that immunization with influenza (flu) vaccines in diabetic patients induces normal or even elevated levels of flu-specific antibodies compared with normal individuals (195–198). However, the ability of the dysfunctional glycated antibodies to neutralize viruses is impaired, which will increase the susceptibility to infections. Farnsworth et al. have shown that in T2DM, class switch defects in the assembly of antibody genes are also present (199).

In a model system, mice with T2DM have decreased amounts of specific anti-*Staphylococcus aureus* antibodies (total as well as IgG), which will increase the risk of infection and morbidity of diabetic mice. However, the levels of IgM were elevated, but inefficient in protecting against infection, possibly because of their inability to directly promote phagocytosis. In another study, Farnsworth et al. have demonstrated that defects in humoral immunity, as shown by decreased levels of total IgG and anti-*Staphylococcus aureus* antibody, aggravate foot infections in a murine model of T2DM (200). This was due to a reduced germinal center induction and decreased numbers of T and B-lymphocytes within the germinal centers. This causes failures in antibody generation and class-switch recombination (200). Mathews et al. have shown that the protective levels of antibodies against *Streptococcus pneumoniae* surface protein A are lower in diabetic patients compared to non-diabetic individuals. These antibodies also have a reduced potential to trigger complement activation on the surface of pneumococci, whereby phagocytosis of the bacteria becomes compromised (201). They showed that hyperglycemia reduces both the antibody titers as well as the ability to deposit complement on the bacteria. The above-mentioned changes in the ability to protect against *S. aureus* and *S. pneumoniae* are important, because these bacteria belong to the most common infection-causing pathogens in diabetic patients. Another major group is constituted by Gram-negative bacteria that commonly cause e.g., urinary tract infections.

#### Cellular Immunity (T-Cells)

Many studies have shown that T-cell functions are impaired in individuals with T2DM (202–205). Elevated levels of activated CD4^+^CD278^+^ T helper cells, cytotoxic T-cells, and Th17 cells have been observed in obese diabetic patients compared to non-obese ones (205, 206). Nevertheless, PBMCs isolated from obese diabetic patients produced smaller amounts of IL-2, IL-6, and TNF-α after stimulation with phytohemagglutinin (PHA) (205). Martinez et al. indicated that diabetic patients have reduced pathogen-specific memory Th17 responses as well as decreased numbers of CD4+ T cells in response to stimulation with *Streptococcus pneumoniae* (206). Th17 cells are critical for the recruitment of neutrophils to the infection site and improve the phagocytosis of invading bacteria and yeast (207).

Moura et al. have shown that diabetic patients, particularly those with foot ulcers, have reduced levels of naive T-cells, but an elevated number of effector T cells and a reduction in the TCR-Vβ repertoire diversity (204). The observed changes are mainly due to an abnormal amount of inflammatory cytokines (e.g., IFN-γ and TNF-α) produced during infection and to subsequent robust stimulation of T-cells. Leung et al. have reported that ischemic tissues of T2DM patients contain elevated numbers of TNF-α and IFN-γ producing Th1 cells but diminished numbers of regulatory T cells (Tregs), which suppress angiogenesis and decrease vascular density (208).

The high rate of infectious diseases in T2DM patients might also be linked to a reduction in the mitochondrial DNA function that causes downstream lymphocyte dysfunction and subsequently increased susceptibility to infection (209–212). In support, we have recently shown that the numbers of IFN-γ producing cells against cytomegalovirus (CMV), Epstein-Barr virus (EBV), and influenza virus are fewer in T2DM patients compared to normal controls (202). Kumar et al. have also investigated the functions of CD8^+^ T cells and NK cells in the whole blood of T2DM patients infected with *Mycobacterium tuberculosis* (M.tb). Compared to controls, the patients exhibited a reduction in cytokine production (IFN-γ, IL-2, IL-17A/F, and TNF-α) and decreased expression of cytotoxic molecules (perforin, granzyme B, and CD107a) (203, 213). These studies conclude that the functions of both CD4^+^ and CD8^+^ T-cell are defective in T2DM patients.

## Effects of T2DM on the Susceptibility of Patients to Infections

T2DM is usually associated with an elevated risk of asymptomatic bacteriuria, urinary tract infections (UTIs), pyelonephritis and non-sexually transmitted genital infections, such as balanitis and vulvovaginal infections (213–215). The incidence of infections with a complicated course is significantly higher in diabetic patients compared to healthy controls (Table 3). It seems that it is principally defects in the innate immune responses of diabetic individuals that are responsible for the increased susceptibility and prevalence of infections (4, 225).

**Table 3:** Dysfunctional immune system in T2DM patients promotes the pathogenesis of infections.

	**Infectious agent**	**References**
**Dysfunctional innate immunity**		
Complement system	*Candida* spp (*albicans, tropicalis, lusitaniae, lipolytica, krusei*), *Streptococcus pneumoniae, Borrelia burgdorferi, and Escherichia coli*	(130–132, 216)
NK cells	*Mycobacterium tuberculosis*	(203, 213)
Neutrophil	*Staphylococcus aureus, Klebsiella pneumoniae*, and *Burkholderia pseudomallei*	(217–221)
Macrophage	*Mycobacterium tuberculosis*	(222, 223)
ILC3	*Mycobacterium tuberculosis*	(224)
**Dysfunctional adaptive immunity**		
B cell (humoral immunity)	*Staphylococcus aureus, Streptococcus pneumoniae*	(199, 201)
CD8^+^Tcells	*Mycobacterium tuberculosis*	(203, 213)

### Bacteria

Thimmappaiah et al. have shown that the cutaneous microbiome is altered among patients with T2DM. Especially dominant is *Staphylococcus epidermidis*, which increases the susceptibility of patients to skin and soft tissue infections (226). Javid et al. have shown that hyperglycemia in diabetic mice makes them more susceptible to the causative pathogen of Lyme disease, *Borrelia burgdorferi* (216). The disease is mainly due to the ability of the bacteria to escape complement opsonization and attack, which leads to an impaired uptake and killing of bacteria by neutrophils (227). Neutrophil dysfunction also increases the susceptibility of diabetic animals to *Staphylococcus aureus* (217), *Klebsiella pneumoniae* (218), and *Burkholderia pseudomallei* (219, 220). Of note, Garnett et al. showed that the treatment of diabetic patients with metformin would reduce hyperglycemia-induced growth of *S. aureus* (228). Hodgson et al. have demonstrated in a mouse model of T2DM that 24 h after a subcutaneous injection of *B. pseudomallei* the expression of IFN-γ, TNF-α, IL-1β, IL-6, and IL-12 cytokines were decreased compared to non-diabetic controls (229). They further demonstrated an excessive polymorphonuclear cell (PMN) infiltration at the site of bacterial injection, unlimited bacterial growth in the spleen and dissemination of bacteria to the lungs of diabetic mice (229). The critical role of neutrophils in resistance against *B. pseudomallei* has been well-documented by Easton et al. (221). However, Buddhisa et al. have demonstrated that in patients with T2DM the expression of programmed cell death ligand 1 (PD-L1) on the surface of *B. pseudomallei* infected neutrophils is increased thus impairing T cell function (230). Kronsteiner et al., have demonstrated that CD3^+^CD4^−^CD8^−^ double-negative T cells and antibodies are important for the survival of diabetic melioidosis patients, while the survival of non-diabetics relies on CD8^+^ T cells and NK cells (231). They also indicated that IFN-γ release from γδ T-cells have an important role in the induction of protective immune responses in diabetic patients.

Deletion of the receptor of AGEs, which is upregulated by elevated levels of AGEs in diabetic hosts, protects diabetic mice from infection with Gram-negative bacteria such as *Acinetobacter baumannii* (232). Asante-Poku et al. have recently demonstrated that T2DM patients, who have active tuberculosis (either caused by *Mycobacterium tuberculosis* or *M. africanum*), are significantly more resistant to therapy compared to patients without diabetes (233). During the progression of T2DM in human subjects, the basal phenotype of macrophages is altered so their capacity to control *Mycobacterium tuberculosis* is diminished (222). Martinez et al. have indicated that alveolar macrophages isolated from diabetic mice express decreased levels of macrophage receptor with collagenous structure (MARCO) and CD14 that are engaged in the recognition of trehalose 6,6'-dimycolate, a bacterial cell wall component (223). Diabetes increases the severity of tuberculosis (TB) and enhances the risk of progression to the active form in latent infections (234, 235). Diabetic TB patients have elevated frequencies of Th1 and Th17 cells as well as increased serum levels of inflammatory cytokines, including IFN-γ, TNF-α, IL-1β, IL-2, IL-6, IL-17A, and IL-18 but decreased levels of IL-22 compared to non-diabetic TB patients. This can contribute to dysfunctional immune responses and poor immune control of a TB infection (236). A positive correlation between the serum levels of IFN-γ, TNF-α, IL-2, and IL-17A with Hb-A1c levels was also observed. This indicates an association between impaired control of diabetes and the proinflammatory milieu. Tripathi et al. have demonstrated that serum levels of IL-22 were significantly decreased in TB-infected T2DM mice and humans compared to non-diabetic TB-infected mice and humans (224). They revealed that the treatment of TB-infected diabetic mice with recombinant IL-22 or ILC3s (cellular source of IL-22) increased the survival of mice, prevented the accumulation of neutrophils near alveoli, diminished the generation of neutrophil elastase 2 (ELA2) and prevented epithelial cell damage (224).

Tan et al. have shown that *B. pseudomallei* and *M. tuberculosis*-infected PBMCs of diabetic patients fail to produce IL-12. This leads to a decreased IFN-γ production, poor bacterial killing and elevated intracellular bacterial loads (237). An impaired IL-12 production is mainly due to decreased intracellular glutathione (GSH) concentrations within the infected cells of diabetic individuals (237). Such a combination of an inflammatory microenvironment and dysfunctional immune responses enhances the bacterial load and can subsequently amplify lung injury and fibrosis in diabetic TB patients. Chellan et al. have further shown that infections caused by *Enterococcus faecalis, Staphylococcus aureus*, and *Pseudomonas aeruginosa* are more prevalent in the wounds of diabetic patients (238). T2DM patients are more susceptible to UTIs caused by antibiotic-resistant *Escherichia coli, Proteus* spp., *Klebsiella* spp., coagulase-negative staphylococci, *Enterobacter* spp., and enterococci (215, 239). Diabetic patients are also more susceptible to *Helicobacter pylori* (*H. pylori*) infections (240).

### Viruses

Cui et al. have recently reported that T2DM patients have an increased risk of infection with Kaposi's sarcoma-associated herpesvirus (KSHV or HHV-8) (241). They further showed that the viral load and antibody titers are positively correlated with blood glucose levels (241). Diabetic patients also have been shown to have an increased risk of infection with the severe acute respiratory syndrome coronavirus (SARS-CoV) (242), Middle East respiratory syndrome coronavirus (MERS-CoV) (243), SARS coronavirus 2 (SARS-CoV-2) (21, 22), hepatitis C virus (HCV) (244–246), and *West Nile virus encephalitis* (WNVE) (247).

Regarding hepatitis infection, Juttada et al. have recently demonstrated that Indian patients with T2DM have a greater prevalence of HBV infection (9.3%) compared to HCV (2.8%) (248). The influenza virus that usually causes self-limiting infections can induce severe forms of the disease in diabetic patients (249, 250). Following the 2009 H1N1 influenza pandemic, diabetic individuals suffered from more severe infections compared to non-diabetic people (251, 252). Diabetic patients have also a higher prevalence of chronic cytomegalovirus (CMV), Herpes simplex virus (especially HSV-1), and varicella-zoster virus infections (253–255). Accordingly, it seems that the immune response against viruses is impaired in diabetics, and these patients need more care during viral infections.

#### Coronaviruses (CoV)

Coronavirus virions are enveloped positive-strand RNA spherical viruses with a diameter of ~125 nm characterized by spike proteins projecting from their surface and with an unusual large RNA genome (256). The spike (S) protein of the virus binds to its receptor on the surface of cells by which intracellular proteases are induced (257–259). Subsequently, the S protein priming and cleavage occurs that allow viral fusion to the plasma membrane and entrance of viral genome into the cells (259). SARS-CoV and SARS-CoV-2 use angiotensin-converting enzyme 2 (ACE2) as their receptor while MERS-CoV uses dipeptidyl peptidase-4 (DPP4) to enter the cells (260, 261). ACE2 is strongly expressed in blood vessels, pancreas, intestine, brain, lungs, heart, and testis (262).

Interestingly, nasal epithelial cells, especially goblet, and ciliated cells express the highest levels of ACE2 and the intracellular protease transmembrane serine protease 2 (TMPRSS2) that facilitates the entrance of the SARS-COV-2 (263). Furthermore, the expression of ACE2 is significantly up-regulated in diabetic patients and those treated with ACE inhibitors (264). Coronaviruses cause respiratory, enteric and central nervous system (CNS) diseases in various animal species except rats and mice (264). Most coronavirus infections are mild, but major outbreaks of deadly pneumonia have been caused by SARS-CoV, MERS-CoV, and SARS-CoV-2 in 2002, 2014, and 2019-2020, respectively (265).

On March 11, 2020, The World Health Organization (WHO) announced the pandemic of SARS-CoV-2, the etiologic agent of coronavirus disease-19 (COVID-19) (265). The novel coronavirus pandemic, which has emanated from Wuhan, China, promotes symptoms similar to those caused by the SARS-CoV outbreak in 2002. The viral pandemic, which has put the world on alert, has caused over 7.9 × 10^6^ confirmed human cases and at least 43 × 10^4^ deaths throughout the world (https://www.worldometers.info/coronavirus/) by June 14, 2020. Most of the infected people experience only mild to moderate respiratory disease and recover soon without the need for special treatment. However, aged individuals and those with health problems, including diabetes, obesity, cardiovascular disease (CVD), hypertension, immune deficiency, and chronic respiratory disease are more likely to develop serious illness (https://www.who.int/health-topics/coronavirus#tab=tab_1). Patients death is mainly due to the acute respiratory distress syndrome, disseminated intravascular coagulation, hemorrhage, coagulopathy, acute organ (e.g., kidney, heart, liver) injury, multi-organ failure, and secondary bacterial infections (266). Elevated levels of adipose-tissue derived adipokines, interferon, and TNF-α in diabetic patients may impair immune-responses against SARS-COV-2 (267, 268). It has been shown that diabetic patients have impaired clearance of SARS-CoV-2 from their circulation (269). Accordingly, diabetic patients due to the diminished viral clearance, impaired T cell function, and accompanied cardiovascular disease are more susceptible to the coronaviruses infection and subsequent cytokine release syndrome (CRS) (270, 271). In support, elevated levels of IL-1β, IL-2, IL-6, IL-7, IL-8, IL-10, IFN-γ, interferon gamma-induced protein 10 (IP-10), granulocyte colony-stimulating factor (G-CSF), macrophage inflammatory protein 1α (MIP1α), serum ferritin, fibrinogen, plasminogen, C-reactive protein (CRP), and D-dimer have been observed in patients with COVID-19 (266, 269, 272, 273). COVID-19 patients, especially those requiring intensive care unit (ICU) have decreased total lymphocytes (lymphopenia), T cells (both CD4+ and CD8+), B cells, and NK cells (274, 275). It should be noted that most of the surviving T cells in such patients have an exhausted phenotype (274). Consequently, disease severity is mainly because of the host immune response to viral infection.

Current evidence about the relationship between pathophysiological mechanisms of diabetes and COVID-19 are limited and further research is still needed.

### Parasites

Patients with T2DM have an elevated risk of infection with *Plasmodium falciparum* (276), *Toxoplasma gondii* (277), *Opisthorchis viverrini* (278), *Strongyloides stercoralis* (279), *Cryptosporidium parvum* (280), *Blastocystis hominis* (281), *Ascaris lumbricoides* (280, 282, 283), and *Giardia lamblia* (283). Interestingly, diabetic patients who were treated with metformin had less *P. falciparum* infections compared to untreated patients (276). Omaña-Molina et al. have shown that in a mouse model of T2DM the animals have an increased susceptibility to granulomatous amoebic encephalitis (GAE) caused by trophozoites of *Acanthamoeba culbertsoni* (284). The possible reasons for the increased risk of diabetics for parasitic infections are metabolic abnormalities and immune dysregulation.

### Fungi

Chellan et al. have shown a higher prevalence of fungal infections in the wounds of diabetic patients (238). The prevalence correlated with the levels of HbA1c. The most widely observed fungal isolates were *C. albicans, Candida parapsilosis, C. tropicalis, Trichosporon asahii*, and *Aspergillus species*. Some of them were resistant to antifungal medications (238). Al Mubarak et al. have also demonstrated that diabetic patients with periodontitis are more susceptible to infection with *C. albicans, C. dubliniensis, C. tropicalis*, and *C. glabrata* (285). The incidence of candidiasis was significantly increased in patients over the age of 40 with HbA1c > 9 (285). It has also been shown that diabetic patients are more susceptible to UTIs caused by *C. albicans* (239).

## Conclusion

Hyperglycemia impairs the normal functions of the circulatory system, gastrointestinal tract, pancreatic beta cells, liver as well as of skeletal muscles to boost systemic insulin resistance. A hyperglycemic environment also leads to immune cells dysfunction. It increases intestinal permeability, which subsequently enhances the risk of infections in T2DM patients. Accordingly, further research is still needed to find missing links between impaired physiological/immunological mechanisms and increased susceptibility to infections in T2DM patients. The information would be important for better therapy and the design of much more effective vaccination strategies in diabetic patients.

## Author Contributions

GD and KK conceived the study and wrote the manuscript. GD contributed to the final revision of the manuscript. MA participated in preparing the first draft. DK and SM were involved in the final revision of the manuscript. All authors contributed to the article and approved the submitted version.

## Conflict of Interest

The authors declare that the research was conducted in the absence of any commercial or financial relationships that could be construed as a potential conflict of interest.

## Abbreviations

AGE: advanced glycation end products
APCs: antigen-presenting cells
CAPCs: circulating angiogenic progenitor cells
CVD: cardiovascular diseases
DCs: dendritic cells
ECs: endothelial cells
EMPs: endothelial microparticles
ER: endoplasmic reticulum
FFAs: free fatty acids
GLUT: glucose transporter
HDLs: high-density lipoproteins
HN: humanin
HUVECs: human umbilical vein endothelial cells
IAPP: Islet amyloid polypeptide
IECs: intestinal epithelial cells
IFN: Interferon
IL: interleukin
MGO: methylglyoxal
MP: microparticle
NETs: Neutrophil Extracellular Traps
NKs: Natural killer cells
NLRP3: nucleotide-binding oligomerization domain, leucine-rich–containing family, pyrin domain-containing 3
Ox-LDL: Oxidized low-density lipoprotein
PPAR: peroxisome proliferator-activated receptors
ROS: reactive oxygen species
sdLDL: small dense LDL
TG: triglyceride
T2DM: type 2 diabetes mellitus
TNF-α: tumor necrosis factor-alpha
UTIs: urinary tract infections.

## References

[1] Moller DE Kaufman KD. Metabolic syndrome: a clinical and molecular perspective. Annu Rev Med. (2005) 56:45–62. 10.1146/annurev.med.56.082103.104751 15660501

[2] Bays HE Toth PP Kris-Etherton PM Abate N Aronne LJ Brown WV. Obesity, adiposity, and dyslipidemia: a consensus statement from the National Lipid Association. J Clin Lipidol. (2013) 7:304–83. 10.1016/j.jacl.2013.04.001 23890517

[3] Lorenzo C Okoloise M Williams K Stern MP Haffner SM. The metabolic syndrome as predictor of type 2 diabetes: the San Antonio heart study. Diabetes Care. (2003) 26:3153–9. 10.2337/diacare.26.11.3153 14578254

[4] Daryabor G Kabelitz D Kalantar K. An update on immune dysregulation in obesity-related insulin resistance. Scand J Immunol. (2019) 89:12747. 10.1111/sji.12747 30593678

[5] Defronzo RA Ferrannini E Groop L Henry RR Herman WH Holst JJ Type 2 diabetes mellitus. Nat Rev Dis Primers. (2015) 1:e15019 10.1038/nrdp.2015.19 27189025

[6] Makowski L Chaib M Rathmell JC. Immunometabolism: from basic mechanisms to translation. Immunol Rev. (2020) 295:5–14. 10.1111/imr.12858 32320073 PMC8056251

[7] Robertson RP Harmon J Tran PO Tanaka Y Takahashi H. Glucose toxicity in beta-cells: type 2 diabetes, good radicals gone bad, and the glutathione connection. Diabetes. (2003) 52:581–7. 10.2337/diabetes.52.3.581 12606496

[8] Folli F Corradi D Fanti P Davalli A Paez A Giaccari A. The role of oxidative stress in the pathogenesis of type 2 diabetes mellitus micro- and macrovascular complications: avenues for a mechanistic-based therapeutic approach. Curr Diabetes Rev. (2011) 7:313–24. 10.2174/157339911797415585 21838680

[9] Zhang Z-Y Miao L-F Qian L-L Wang N Qi M-M Zhang Y-M. Molecular mechanisms of glucose fluctuations on diabetic complications. Front Endocrinol. (2019) 10:640. 10.3389/fendo.2019.00640 31620092 PMC6759481

[10] Cui H Kong Y Zhang H. Oxidative stress, mitochondrial dysfunction, and aging. J Signal Transduct. (2012) 2012:e646354. 10.1155/2012/646354 21977319 PMC3184498

[11] Lowell BB Shulman GI. Mitochondrial dysfunction and type 2 diabetes. Science. (2005) 307:384–7. 10.1126/science.1104343 15662004

[12] Pinti MV Fink GK Hathaway QA Durr AJ Kunovac A Hollander JM. Mitochondrial dysfunction in type 2 diabetes mellitus: an organ-based analysis. Am J Physiol Endocrinol Metab. (2019) 316:E268–85. 10.1152/ajpendo.00314.2018 30601700 PMC6397358

[13] Yuan T Yang T Chen H Fu D Hu Y Wang J. New insights into oxidative stress and inflammation during diabetes mellitus-accelerated atherosclerosis. Redox Biol. (2019) 20:247–60. 10.1016/j.redox.2018.09.025 30384259 PMC6205410

[14] Singh VP Bali A Singh N Jaggi AS. Advanced glycation end products and diabetic complications. Korean J Physiol Pharmacol. (2014) 18:1–14. 10.4196/kjpp.2014.18.1.1 24634591 PMC3951818

[15] Maessen DE Stehouwer CD Schalkwijk CG. The role of methylglyoxal and the glyoxalase system in diabetes and other age-related diseases. Clin Sci. (2015) 128:839–61. 10.1042/CS20140683 25818485

[16] Hegab Z Gibbons S Neyses L Mamas MA. Role of advanced glycation end products in cardiovascular disease. World J Cardiol. (2012) 4:90–102. 10.4330/wjc.v4.i4.90 22558488 PMC3342583

[17] Davis KE Prasad C Vijayagopal P Juma S Imrhan V. Advanced glycation end products, inflammation, and chronic metabolic diseases: links in a chain? Crit Rev Food Sci Nutr. (2016) 56:989–98. 10.1080/10408398.2012.744738 25259686

[18] Ramasamy R Yan SF Herold K Clynes R Schmidt AM. Receptor for advanced glycation end products: fundamental roles in the inflammatory response: winding the way to the pathogenesis of endothelial dysfunction and atherosclerosis. Ann N Y Acad Sci. (2008) 1126:7–13. 10.1196/annals.1433.056 18448789 PMC3049155

[19] Gurung M Li Z You H Rodrigues R Jump DB Morgun A. Role of gut microbiota in type 2 diabetes pathophysiology. EBiomedicine. (2020) 51:102590. 10.1016/j.ebiom.2019.11.051 31901868 PMC6948163

[20] Carey IM Critchley JA Dewilde S Harris T Hosking FJ Cook DG. Risk of infection in type 1 and type 2 diabetes compared with the general population: a matched cohort study. Diabetes Care. (2018) 41:513–21. 10.2337/dc17-2131 29330152

[21] Wu Z Mcgoogan JM. Characteristics of and important lessons from the coronavirus disease 2019 (COVID-19) outbreak in china: summary of a report of 72 314 cases from the chinese center for disease control and prevention. JAMA. (2020) 323:1239–42. 10.1001/jama.2020.2648. 32091533

[22] Zhou F Yu T Du R Fan G Liu Y Liu Z Clinical course and risk factors for mortality of adult inpatients with COVID-19 in Wuhan, China: a retrospective cohort study. Lancet. (2020) 395:1054–62. 10.1016/S0140-6736(20)30566-3 32171076 PMC7270627

[23] Pober JS Sessa WC. Evolving functions of endothelial cells in inflammation. Nat Rev Immunol. (2007) 7:803–15. 10.1038/nri2171 17893694

[24] Tran-Dinh A Diallo D Delbosc S Varela-Perez LM Dang QB Lapergue B. HDL and endothelial protection. Br J Pharmacol. (2013) 169:493–511. 10.1111/bph.12174 23488589 PMC3682699

[25] Almdal T Scharling H Jensen JS Vestergaard H. The independent effect of type 2 diabetes mellitus on ischemic heart disease, stroke, and death: a population-based study of 13,000 men and women with 20 years of follow-up. Arch Intern Med. (2004) 164:1422–6. 10.1001/archinte.164.13.1422 15249351

[26] Schalkwijk CG Stehouwer CD. Vascular complications in diabetes mellitus: the role of endothelial dysfunction. Clin Sci. (2005) 109:143–59. 10.1042/CS20050025 16033329

[27] Low Wang Cecilia C Hess Connie N Hiatt William R Goldfine Allison B. Clinical update: cardiovascular disease in diabetes mellitus. Circulation. (2016) 133:2459–502. 10.1161/CIRCULATIONAHA.116.022194 27297342 PMC4910510

[28] Irshad Z Xue M Ashour A Larkin JR Thornalley PJ Rabbani N. Activation of the unfolded protein response in high glucose treated endothelial cells is mediated by methylglyoxal. Sci Rep. (2019) 9:7889. 10.1038/s41598-019-44358-1 31133647 PMC6536510

[29] Domingo-Espín J Nilsson O Bernfur K Del Giudice R Lagerstedt JO. Site-specific glycations of apolipoprotein A-I lead to differentiated functional effects on lipid-binding and on glucose metabolism. Biochim Biophys Acta Mol Basis Dis. (2018) 1864:2822–34. 10.1016/j.bbadis.2018.05.014 29802959

[30] Liu D Ji L Zhao M Wang Y Guo Y Li L. Lysine glycation of apolipoprotein A-I impairs its anti-inflammatory function in type 2 diabetes mellitus. J Mol Cell Cardiol. (2018) 122:47–57. 10.1016/j.yjmcc.2018.08.001 30092227

[31] Vaisar T Couzens E Hwang A Russell M Barlow CE Defina LF. Type 2 diabetes is associated with loss of HDL endothelium protective functions. PLoS ONE. (2018) 13:e0192616. 10.1371/journal.pone.0192616 29543843 PMC5854245

[32] Mcclung JA Naseer N Saleem M Rossi GP Weiss MB Abraham NG. Circulating endothelial cells are elevated in patients with type 2 diabetes mellitus independently of HbA(1)c. Diabetologia. (2005) 48:345–50. 10.1007/s00125-004-1647-5 15660261

[33] Libby P Buring JE Badimon L Hansson GK Deanfield J Bittencourt MS Atherosclerosis. Nat Rev Dis Primers. (2019) 5:e56 10.1038/s41572-019-0106-z 31420554

[34] Mooradian AD. Dyslipidemia in type 2 diabetes mellitus. Nat Clin Pract Endocrinol Metab. (2009) 5:150–9. 10.1038/ncpendmet1066 19229235

[35] El-Seweidy MM Sarhan Amin R Husseini Atteia H El-Zeiky RR Al-Gabri NA. Dyslipidemia induced inflammatory status, platelet activation and endothelial dysfunction in rabbits: Protective role of 10-dehydrogingerdione. Biomed Pharmacother. (2019) 110:456–64. 10.1016/j.biopha.2018.11.140 30530048

[36] Fowler MJ Microvascular and macrovascular complications of diabetes. Clin Diabetes. (2008) 26:77–82. 10.2337/diaclin.26.2.77

[37] Shimada K Mokuno H Matsunaga E Miyazaki T Sumiyoshi K Kume A. Predictive value of circulating oxidized ldl for cardiac events in type 2 diabetic patients with coronary artery disease. Diabetes Care. (2004) 27:843–4. 10.2337/diacare.27.3.843 14988319

[38] Ivanova EA Myasoedova VA Melnichenko AA Grechko AV Orekhov AN. Small dense low-density lipoprotein as biomarker for atherosclerotic diseases. Oxid Med Cell Longev. (2017) 2017:1273042. 10.1155/2017/1273042 28572872 PMC5441126

[39] Cominacini L Pasini AF Garbin U Davoli A Tosetti ML Campagnola M. Oxidized low density lipoprotein (ox-LDL) binding to ox-LDL receptor-1 in endothelial cells induces the activation of NF-kappa B through an increased production of intracellular reactive oxygen species. J Biol Chem. (2000) 275:12633–8. 10.1074/jbc.275.17.12633 10777555

[40] Chen M Masaki T Sawamura T. LOX-1, the receptor for oxidized low-density lipoprotein identified from endothelial cells: implications in endothelial dysfunction and atherosclerosis. Pharmacol Ther. (2002) 95:89–100. 10.1016/S0163-7258(02)00236-X 12163130

[41] Moore KJ Sheedy FJ Fisher EA. Macrophages in atherosclerosis: a dynamic balance. Nat Rev Immunol. (2013) 13:709–21. 10.1038/nri3520 23995626 PMC4357520

[42] Flynn MC Pernes G Lee MKS Nagareddy PR Murphy AJ. Monocytes, macrophages, and metabolic disease in atherosclerosis. Front Pharmacol. (2019) 10:666. 10.3389/fphar.2019.00666 31249530 PMC6584106

[43] Chistiakov DA Bobryshev YV Orekhov AN. Macrophage-mediated cholesterol handling in atherosclerosis. J Cell Mol Med. (2016) 20:17–28. 10.1111/jcmm.12689 26493158 PMC4717859

[44] Zafar N Krishnasamy SS Shah J Rai SN Riggs DW Bhatnagar A. Circulating angiogenic stem cells in type 2 diabetes are associated with glycemic control and endothelial dysfunction. PLoS ONE. (2018) 13:e0205851. 10.1371/journal.pone.0205851 30321232 PMC6188890

[45] Kahn MB Yuldasheva NY Cubbon RM Smith J Rashid ST Viswambharan H. Insulin resistance impairs circulating angiogenic progenitor cell function and delays endothelial regeneration. Diabetes. (2011) 60:1295–303. 10.2337/db10-1080 21317296 PMC3064103

[46] Vigorelli V Resta J Bianchessi V Lauri A Bassetti B Agrifoglio M. Abnormal DNA methylation induced by hyperglycemia reduces CXCR 4 gene expression in CD 34(+) stem cells. J Am Heart Assoc. (2019) 8:e010012. 10.1161/JAHA.118.010012 31018749 PMC6512087

[47] Cappellari R D'anna M Menegazzo L Bonora BM Albiero M Avogaro A Diabetes mellitus impairs circulating proangiogenic granulocytes. Diabetologia. (2020). 10.1007/s00125-020-05142-3. [Epub ahead of print]. 32306097

[48] Ott C Jacobs K Haucke E Navarrete Santos A Grune T Simm A. Role of advanced glycation end products in cellular signaling. Redox Biol. (2014) 2:411–29. 10.1016/j.redox.2013.12.016 24624331 PMC3949097

[49] Ren X Ren L Wei Q Shao H Chen L Liu N. Advanced glycation end-products decreases expression of endothelial nitric oxide synthase through oxidative stress in human coronary artery endothelial cells. Cardiovasc Diabetol. (2017) 16:52. 10.1186/s12933-017-0531-9 28427390 PMC5397770

[50] Lan KC Chiu CY Kao CW Huang KH Wang CC Huang KT. Advanced glycation end-products induce apoptosis in pancreatic islet endothelial cells via NF-kappaB-activated cyclooxygenase-2/prostaglandin E2 up-regulation. PLoS ONE. (2015) 10:e0124418. 10.1371/journal.pone.0124418 25898207 PMC4405342

[51] Zhou Q Cheng K-W Gong J Li ETS Wang M. Apigenin and its methylglyoxal-adduct inhibit advanced glycation end products-induced oxidative stress and inflammation in endothelial cells. Biochem Pharmacol. (2019) 166:231–41. 10.1016/j.bcp.2019.05.027 31158339

[52] Chettab K Zibara K Belaiba SR Mcgregor JL. Acute hyperglycaemia induces changes in the transcription levels of 4 major genes in human endothelial cells: macroarrays-based expression analysis. Thromb Haemost. (2002) 87:141–8. 10.1055/s-0037-1612957 11848444

[53] Bammert TD Hijmans JG Reiakvam WR Levy MV Brewster LM Goldthwaite ZA. High glucose derived endothelial microparticles increase active caspase-3 and reduce microRNA-Let-7a expression in endothelial cells. Biochem Biophys Res Commun. (2017) 493:1026–9. 10.1016/j.bbrc.2017.09.098 28942148 PMC5652070

[54] Jansen F Wang H Przybilla D Franklin BS Dolf A Pfeifer P. Vascular endothelial microparticles-incorporated microRNAs are altered in patients with diabetes mellitus. Cardiovasc Diabetol. (2016) 15:49. 10.1186/s12933-016-0367-8 27005938 PMC4804519

[55] Ho FM Lin WW Chen BC Chao CM Yang CR Lin LY. High glucose-induced apoptosis in human vascular endothelial cells is mediated through NF-kappaB and c-Jun NH2-terminal kinase pathway and prevented by PI3K/Akt/eNOS pathway. Cell Signal. (2006) 18:391–9. 10.1016/j.cellsig.2005.05.009 15970429

[56] Su J An X-R Li Q Li X-X Cong X-D Xu M. Improvement of vascular dysfunction by argirein through inhibiting endothelial cell apoptosis associated with ET-1/Nox4 signal pathway in diabetic rats. Sci Rep. (2018) 8:12620. 10.1038/s41598-018-30386-w 30135489 PMC6105644

[57] Dang L Seale JP Qu X. High glucose-induced human umbilical vein endothelial cell hyperpermeability is dependent on protein kinase C activation and independent of the Ca^2+^-nitric oxide signalling pathway. Clin Exp Pharmacol Physiol. (2005) 32:771–6. 10.1111/j.1440-1681.2005.04266.x 16173935

[58] Zhao X-Y Wang X-F Li L Zhang L Shen D-L Li D-H. Effects of high glucose on human umbilical vein endothelial cell permeability and myosin light chain phosphorylation. Diabetol Metab Syndr. (2015) 7:98. 10.1186/s13098-015-0098-0 26583048 PMC4650340

[59] Hassanpour M Rezabakhsh A Rahbarghazi R Nourazarian A Nouri M Avci ÇB. Functional convergence of Akt protein with VEGFR-1 in human endothelial progenitor cells exposed to sera from patient with type 2 diabetes mellitus. Microvasc Res. (2017) 114:101–13. 10.1016/j.mvr.2017.07.002 28732797

[60] Lee C Yen K Cohen P. Humanin: a harbinger of mitochondrial-derived peptides? Trends Endocrinol Metab. (2013) 24:222–8. 10.1016/j.tem.2013.01.005 23402768 PMC3641182

[61] Klein LE Cui L Gong Z Su K Muzumdar R. A humanin analog decreases oxidative stress and preserves mitochondrial integrity in cardiac myoblasts. Biochem Biophys Res Commun. (2013) 440:197–203. 10.1016/j.bbrc.2013.08.055 23985350 PMC3853355

[62] Wang X Wu Z He Y Zhang H Tian L Zheng C. Humanin prevents high glucose-induced monocyte adhesion to endothelial cells by targeting KLF2. Mol Immunol. (2018) 101:245–50. 10.1016/j.molimm.2018.07.008 30029058

[63] Grozovsky R Hoffmeister KM Falet H. Novel clearance mechanisms of platelets. Curr Opin Hematol. (2010) 17:585–9. 10.1097/MOH.0b013e32833e7561 20729731 PMC4303238

[64] Soma P Swanepoel AC Du Plooy JN Mqoco T Pretorius E. Flow cytometric analysis of platelets type 2 diabetes mellitus reveals ‘angry’ platelets. Cardiovasc Diabetol. (2016) 15:52. 10.1186/s12933-016-0373-x 27036108 PMC4818425

[65] Pretorius L Thomson GJA Adams RCM Nell TA Laubscher WA Pretorius E. Platelet activity and hypercoagulation in type 2 diabetes. Cardiovasc Diabetol. (2018) 17:141. 10.1186/s12933-018-0783-z 30388964 PMC6214175

[66] Santilli F Liani R Di Fulvio P Formoso G Simeone P Tripaldi R. Increased circulating resistin is associated with insulin resistance, oxidative stress and platelet activation in type 2 diabetes mellitus. Thromb Haemost. (2016) 116:1089–99. 10.1160/TH16-06-0471 27709225

[67] Steiner M Reinhardt KM Krammer B Ernst B Blann AD. Increased levels of soluble adhesion molecules in type 2 (non-insulin dependent) diabetes mellitus are independent of glycaemic control. Thromb Haemost. (1994) 72:979–84. 10.1055/s-0038-1648993 7537916

[68] Benameur T Osman A Parray A Ait Hssain A Munusamy S Agouni A Molecular mechanisms underpinning microparticle-mediated cellular injury in cardiovascular complications associated with diabetes. Oxid Med Cell Longev. (2019) 2019:6475187 10.1155/2019/6475187 30915196 PMC6399542

[69] Inoue H Saito M Kouchi K Asahara S-I Nakamura F Kido Y. Association between mean platelet volume in the pathogenesis of type 2 diabetes mellitus and diabetic macrovascular complications in Japanese patients. J Diabetes Invest.(2019) 12:13198 10.1111/jdi.13198 31833219 PMC7378450

[70] Gkaliagkousi E Nikolaidou B Gavriilaki E Lazaridis A Yiannaki E Anyfanti P. Increased erythrocyte- and platelet-derived microvesicles in newly diagnosed type 2 diabetes mellitus. Diab Vasc Dis Res. (2019) 16:458–65. 10.1177/1479164119844691 31046456

[71] Martín-Timón I Sevillano-Collantes C Segura-Galindo A Del Cañizo-Gómez FJ. Type 2 diabetes and cardiovascular disease: have all risk factors the same strength? World J Diabetes. (2014) 5:444–70. 10.4239/wjd.v5.i4.444 25126392 PMC4127581

[72] Zhu W Li W Silverstein RL. Advanced glycation end products induce a prothrombotic phenotype in mice via interaction with platelet CD36. Blood. (2012) 119:6136–44. 10.1182/blood-2011-10-387506 22431576 PMC3383021

[73] Dorsam RT Kunapuli SP. Central role of the P2Y12 receptor in platelet activation. J Clin Invest. (2004) 113:340–5. 10.1172/JCI20986 14755328 PMC324551

[74] Zhou M Gao M Luo Y Gui R Ji H. Long non-coding RNA metallothionein 1 pseudogene 3 promotes p2y12 expression by sponging miR-126 to activate platelet in diabetic animal model. Platelets. (2019) 30:452–9. 10.1080/09537104.2018.1457781 29617185

[75] Thursby E Juge N. Introduction to the human gut microbiota. Biochem J. (2017) 474:1823–36. 10.1042/BCJ20160510 28512250 PMC5433529

[76] Thaiss CA Levy M Grosheva I Zheng D Soffer E Blacher E. Hyperglycemia drives intestinal barrier dysfunction and risk for enteric infection. Science. (2018) 259:1376–83. 10.1126/science.aar3318 29519916

[77] Xu J Liang R Zhang W Tian K Li J Chen X. *Faecalibacterium prausnitzii*-derived microbial anti-inflammatory molecule regulates intestinal integrity in diabetes mellitus mice via modulating tight junction protein expression. J Diabetes. (2020) 12:224–36. 10.1111/1753-0407.12986 31503404 PMC7064962

[78] Adeshirlarijaney A Gewirtz AT. Considering gut microbiota in treatment of type 2 diabetes mellitus. Gut Microbes. (2020) 11:253–64. 10.1080/19490976.2020.1717719 32005089 PMC7524291

[79] Ouyang J Isnard S Lin J Fombuena B Marette A Routy B. Metformin effect on gut microbiota: insights for HIV-related inflammation. AIDS Res Ther. (2020) 17:10. 10.1186/s12981-020-00267-2 32156291 PMC7063824

[80] Ouyang J Lin J Isnard S Fombuena B Peng X Marette A. The bacterium *Akkermansia muciniphila*: a sentinel for gut permeability and its relevance to HIV-related inflammation. Front Immunol. (2020) 11:645. 10.3389/fimmu.2020.00645 32328074 PMC7160922

[81] Powell LA Warpeha KM Xu W Walker B Trimble ER. High glucose decreases intracellular glutathione concentrations and upregulates inducible nitric oxide synthase gene expression in intestinal epithelial cells. J Mol Endocrinol. (2004) 33:797–803. 10.1677/jme.1.01671 15591036

[82] Swaminathan S Fonseca VA Alam MG Shah SV. The role of iron in diabetes and its complications. Diabetes Care. (2007) 30:1926–33. 10.2337/dc06-2625 17429063

[83] Zhao L Bartnikas T Chu X Klein J Yun C Srinivasan S Hyperglycemia promotes microvillus membrane expression of DMT1 in intestinal epithelial cells in a PKCalpha-dependent manner. FASEB J. (2018) 33:3549–61. 10.1096/fj.201801855R 30423260 PMC6404579

[84] Butler AE Janson J Bonner-Weir S Ritzel R Rizza RA Butler PC Beta-cell deficit and increased beta-cell apoptosis in humans with type 2 diabetes. Diabetes. (2003) 52:102–10. 10.2337/diabetes.52.1.102 12502499

[85] Oh YS Bae GD Baek DJ Park E-Y Jun H-S. Fatty acid-induced lipotoxicity in pancreatic beta-cells during development of type 2 diabetes. Front Endocrinol. (2018) 9:384. 10.3389/fendo.2018.00384 30061862 PMC6054968

[86] Zang L Hao H Liu J Li Y Han W Mu Y Mesenchymal stem cell therapy in type 2 diabetes mellitus. Diabetol Metab Syndr. (2017) 9:36 10.1186/s13098-017-0233-1 28515792 PMC5433043

[87] Rattananinsruang P Dechsukhum C Leeanansaksiri W. Establishment of insulin-producing cells from human embryonic stem cells underhypoxic condition for cell based therapy. Front Cell Dev Biol. (2018) 6:49. 10.3389/fcell.2018.00049 29868580 PMC5962719

[88] Sun Y Shi H Yin S Ji C Zhang X Zhang B. Human mesenchymal stem cell derived exosomes alleviate type 2 diabetes mellitus by reversing peripheral insulin resistance and relieving β-cell destruction. ACS Nano. (2018) 12:7613–28. 10.1021/acsnano.7b07643 30052036

[89] Daryabor G Shiri EH Kamali-Sarvestani E. A simple method for the generation of insulin producing cells from bone marrow mesenchymal stem cells. In Vitro Cell Dev Biol Anim. (2019) 55:462–71. 10.1007/s11626-019-00358-z 31111346

[90] Yang SY Lee JJ Lee JH Lee K Oh SH Lim YM. Secretagogin affects insulin secretion in pancreatic beta-cells by regulating actin dynamics and focal adhesion. Biochem J. (2016) 473:1791–803. 10.1042/BCJ20160137 27095850 PMC4901359

[91] Sharma AK Khandelwal R Kumar MJM Ram NS Chidananda AH Raj TA. Secretagogin regulates insulin signaling by direct insulin binding. iScience. (2019) 21:736–53. 10.1016/j.isci.2019.10.066 31734536 PMC6864339

[92] Hansson SF Zhou A-X Vachet P Eriksson JW Pereira MJ Skrtic S. Secretagogin is increased in plasma from type 2 diabetes patients and potentially reflects stress and islet dysfunction. PLoS ONE. (2018) 13:e0196601. 10.1371/journal.pone.0196601 29702679 PMC5922551

[93] Westermark P Andersson A Westermark GT. Islet amyloid polypeptide, islet amyloid, and diabetes mellitus. Physiol Rev. (2011) 91:795–826. 10.1152/physrev.00042.2009 21742788

[94] Fernandez MS. Human IAPP amyloidogenic properties and pancreatic beta-cell death. Cell Calcium. (2014) 56:416–27. 10.1016/j.ceca.2014.08.011 25224501

[95] Ribeiro D Horvath I Heath N Hicks R Forslöw A Wittung-Stafshede P. Extracellular vesicles from human pancreatic islets suppress human islet amyloid polypeptide amyloid formation. Proc Natl Acad Sci USA. (2017) 114:11127–32. 10.1073/pnas.1711389114 28973954 PMC5651775

[96] Chatterjee Bhowmick D Jeremic A. Functional proteasome complex is required for turnover of islet amyloid polypeptide in pancreatic beta-cells. J Biol Chem. (2018) 293:14210–23. 10.1074/jbc.RA118.002414 30012886 PMC6139561

[97] Mo X-D Gao L-P Wang Q-J Yin J Jing Y-H. Lipid accelerating the fibril of islet amyloid polypeptide aggravated the pancreatic islet injury *in vitro* and *in vivo*. Lipids Health Dis. (2018) 17:42. 10.1186/s12944-018-0694-8 29523142 PMC5845206

[98] Dhar A Dhar I Jiang B Desai KM Wu L. Chronic methylglyoxal infusion by minipump causes pancreatic beta-cell dysfunction and induces type 2 diabetes in Sprague-Dawley rats. Diabetes. (2011) 60:899–908. 10.2337/db10-0627 21300844 PMC3046851

[99] Wang H Meng QH Gordon JR Khandwala H Wu L. Proinflammatory and proapoptotic effects of methylglyoxal on neutrophils from patients with type 2 diabetes mellitus. Clin Biochem. (2007) 40:1232–9. 10.1016/j.clinbiochem.2007.07.016 17825811

[100] Bo J Xie S Guo Y Zhang C Guan Y Li C. Methylglyoxal impairs insulin secretion of pancreatic β-cells through increased production of ROS and mitochondrial dysfunction mediated by upregulation of UCP2 and MAPKs. J Diabetes Res. (2016) 2016:2029854. 10.1155/2016/2029854 26779540 PMC4686727

[101] Cinti F Bouchi R Kim-Muller JY Ohmura Y Sandoval PR Masini M. Evidence of beta-cell dedifferentiation in human type 2 diabetes. J Clin Endocrinol Metab. (2016) 101:1044–54. 10.1210/jc.2015-2860 26713822 PMC4803182

[102] Sinha RA Singh BK Yen PM. Direct effects of thyroid hormones on hepatic lipid metabolism. Nat Rev Endocrinol. (2018) 14:259–69. 10.1038/nrendo.2018.10 29472712 PMC6013028

[103] Leite NC Salles GF Araujo ALE Villela-Nogueira CA Cardoso CRL Prevalence and associated factors of non-alcoholic fatty liver disease in patients with type-2 diabetes mellitus. Liver Int. (2009) 29:113–9. 10.1111/j.1478-3231.2008.01718.x 18384521

[104] Wu H Zhang T Pan F Steer CJ Li Z Chen X. MicroRNA-206 prevents hepatosteatosis and hyperglycemia by facilitating insulin signaling and impairing lipogenesis. J Hepatol. (2017) 66:816–24. 10.1016/j.jhep.2016.12.016 28025059 PMC5568011

[105] Senfeld JI Shen J Evidence for P2Y2 receptor facilitation of hyperglycemia-induced insulin resistance in human hepatocytes. FASEB J. (2020) 34:1 10.1096/fasebj.2020.34.s1.03029

[106] Michelotti GA Machado MV Diehl AM. NAFLD, NASH and liver cancer. Nat Rev Gastroenterol Hepatol. (2013) 10:656–65. 10.1038/nrgastro.2013.183 24080776

[107] Dang S-Y Leng Y Wang Z-X Xiao X Zhang X Wen T. Exosomal transfer of obesity adipose tissue for decreased miR-141-3p mediate insulin resistance of hepatocytes. Int J Biol Sci. (2019) 15:351–68. 10.7150/ijbs.28522 30745826 PMC6367552

[108] Heo YJ Choi S-E Jeon JY Han SJ Kim DJ Kang Y. Visfatin induces inflammation and insulin resistance via the NFκb and STAT3 signaling pathways in hepatocytes. J Diabetes Res. (2019) 2019:4021623. 10.1155/2019/4021623 31396538 PMC6664505

[109] Jing Y Sun Q Xiong X Meng R Tang S Cao S. Hepatocyte growth factor alleviates hepatic insulin resistance and lipid accumulation in high-fat diet-fed mice. J Diabetes Investig. (2019) 10:251–60. 10.1111/jdi.12904 30070033 PMC6400203

[110] Taylor R. Pathogenesis of type 2 diabetes: tracing the reverse route from cure to cause. Diabetologia. (2008) 51:1781–9. 10.1007/s00125-008-1116-7 18726585

[111] Wu C Xu G Tsai S-YA Freed WJ Lee C-T. Transcriptional profiles of type 2 diabetes in human skeletal muscle reveal insulin resistance, metabolic defects, apoptosis, and molecular signatures of immune activation in response to infections. Biochem Biophys Res Commun. (2017) 482:282–8. 10.1016/j.bbrc.2016.11.055 27847319

[112] Khan IM Perrard XY Brunner G Lui H Sparks LM Smith SR. Intermuscular and perimuscular fat expansion in obesity correlates with skeletal muscle T cell and macrophage infiltration and insulin resistance. Int J Obes. (2015) 39:1607–18. 10.1038/ijo.2015.104 26041698 PMC5007876

[113] Ciaraldi TP Ryan AJ Mudaliar SR Henry RR. Altered myokine secretion is an intrinsic property of skeletal muscle in type 2 diabetes. PLoS ONE. (2016) 11:e0158209. 10.1371/journal.pone.0158209 27453994 PMC4959771

[114] Pinto-Junior DC Silva KS Michalani ML Yonamine CY Esteves JV Fabre NT. Advanced glycation end products-induced insulin resistance involves repression of skeletal muscle GLUT4 expression. Sci Rep. (2018) 8:8109. 10.1038/s41598-018-26482-6 29802324 PMC5970140

[115] Mowat AG Baum J. Chemotaxis of polymorphonuclear leukocytes from patients with diabetes mellitus. N Engl J Med. (1971) 284:621–7. 10.1056/NEJM197103252841201 5545603

[116] Delamaire M Maugendre D Moreno M Le Goff MC Allannic H Genetet B. Impaired leucocyte functions in diabetic patients. Diabetic Med. (1997). 14:29–34. 9017350 10.1002/(SICI)1096-9136(199701)14:1<29::AID-DIA300>3.0.CO;2-V

[117] Joshi N Caputo GM Weitekamp MR Karchmer A. Infections in patients with diabetes mellitus. N Engl J Med. (1999) 341:1906–12. 10.1056/NEJM199912163412507 10601511

[118] Rubinstein R Genaro A Motta A Cremaschi G Wald M. Impaired immune responses in streptozotocin-induced type I diabetes in mice. Involvement of high glucose. Clin Exp Immunol. (2008) 154:235–46. 10.1111/j.1365-2249.2008.03742.x 18778365 PMC2612711

[119] Casqueiro J Casqueiro J Alves C. Infections in patients with diabetes mellitus: a review of pathogenesis. Indian J Endocrinol Metab. (2012) 16:S27–36. 10.4103/2230-8210.94253 22701840 PMC3354930

[120] Rubinstein M Genaro A Wald M. Differential effect of hyperglycaemia on the immune response in an experimental model of diabetes in BALB/cByJ and C57Bl/6J mice: participation of oxidative stress. Clin Exp Immunol. (2013) 171:319–29. 10.1111/cei.12020 23379439 PMC3569540

[121] Donath MY Shoelson SE. Type 2 diabetes as an inflammatory disease. Nat Rev Immunol. (2011) 11:98–107. 10.1038/nri2925 21233852

[122] Atashzar MR Daryabor G Kabelitz D Kalantar K. Pyrin and hematopoietic interferon-inducible nuclear protein domain proteins: innate immune sensors for cytosolic and nuclear DNA. Crit Rev Immunol. (2019) 39:275–88. 10.1615/CritRevImmunol.2020033114 32421969

[123] Fang X Dorcely B Ding X-P Yin S Son N-H Hu S-L. Glycemic reduction alters white blood cell counts and inflammatory gene expression in diabetes. J Diabetes Compl. (2018) 32:1027–34. 10.1016/j.jdiacomp.2018.08.003 30197161 PMC6174091

[124] De Souza Prestes A Dos Santos MM Ecker A De Macedo GT Fachinetto R Bressan GN. Methylglyoxal disturbs the expression of antioxidant, apoptotic and glycation responsive genes and triggers programmed cell death in human leukocytes. Toxicol in Vitro. (2019) 55:33–42. 10.1016/j.tiv.2018.11.001 30447388

[125] Hu R Xia CQ Butfiloski E Clare-Salzler M. Effect of high glucose on cytokine production by human peripheral blood immune cells and type I interferon signaling in monocytes: Implications for the role of hyperglycemia in the diabetes inflammatory process and host defense against infection. Clin Immunol. (2018) 195:139–48. 10.1016/j.clim.2018.06.003 29894743 PMC6119493

[126] Hu Z Ma C Liang Y Zou S Liu X. Osteoclasts in bone regeneration under type 2 diabetes mellitus. Acta Biomater. (2019) 84:402–13. 10.1016/j.actbio.2018.11.052 30508657 PMC6326860

[127] Gan Y-H. Host susceptibility factors to bacterial infections in type 2 diabetes. PLoS Pathog. (2013) 9:e1003794. 10.1371/journal.ppat.1003794 24385898 PMC3873456

[128] Ricklin D Hajishengallis G Yang K Lambris JD. Complement: a key system for immune surveillance and homeostasis. Nat Immunol. (2010) 11:785–97. 10.1038/ni.1923 20720586 PMC2924908

[129] Ilyas R Wallis R Soilleux EJ Townsend P Zehnder D Tan BK. High glucose disrupts oligosaccharide recognition function via competitive inhibition: a potential mechanism for immune dysregulation in diabetes mellitus. Immunobiology. (2011) 216:126–31. 10.1016/j.imbio.2010.06.002 20674073 PMC3088832

[130] Barkai LJ Sipter E Csuka D Prohaszka Z Pilely K Garred P. Decreased ficolin-3-mediated complement lectin pathway activation and alternative pathway amplification during bacterial infections in patients with type 2 diabetes mellitus. Front Immunol. (2019) 10:509. 10.3389/fimmu.2019.00509 30949171 PMC6436462

[131] Patel PN Sah P Chandrashekar C Vidyasagar S Venkata Rao J Tiwari M. Oral candidal speciation, virulence and antifungal susceptibility in type 2 diabetes mellitus. Diabetes Res Clin Pract. (2017) 125:10–9. 10.1016/j.diabres.2017.01.001 28131069

[132] Jhugroo C Divakar DD Jhugroo P Al-Amri SAS Alahmari AD Vijaykumar S. Characterization of oral mucosa lesions and prevalence of yeasts in diabetic patients: a comparative study. Microb Pathog. (2019). 126:363–7. 10.1016/j.micpath.2018.11.028 30471434

[133] Chikazawa M Shibata T Hatasa Y Hirose S Otaki N Nakashima F. Identification of C1q as a binding protein for advanced glycation end products. Biochemistry. (2016) 55:435–46. 10.1021/acs.biochem.5b00777 26731343

[134] Qin X Goldfine A Krumrei N Grubissich L Acosta J Chorev M. Glycation inactivation of the complement regulatory protein CD59: a possible role in the pathogenesis of the vascular complications of human diabetes. Diabetes. (2004) 53:2653–61. 10.2337/diabetes.53.10.2653 15448097

[135] Bus P Chua JS Klessens CQF Zandbergen M Wolterbeek R Van Kooten C. Complement activation in patients with diabetic nephropathy. Kidney Int Rep. (2018) 3:302–13. 10.1016/j.ekir.2017.10.005 29725633 PMC5932121

[136] Liu K Nussenzweig MC. Origin and development of dendritic cells. Immunol Rev. (2010) 234:45–54. 10.1111/j.0105-2896.2009.00879.x 20193011

[137] Watowich SS Liu YJ. Mechanisms regulating dendritic cell specification and development. Immunol Rev. (2010) 238:76–92. 10.1111/j.1600-065X.2010.00949.x 20969586 PMC3039024

[138] Hinkmann C Knerr I Hahn EG Lohmann T Seifarth CC. Reduced frequency of peripheral plasmacytoid dendritic cells in type 1 diabetes. Horm Metab Res. (2008) 40:767–71. 10.1055/s-2008-1080896 18622893

[139] Seifarth CC Hinkmann C Hahn EG Lohmann T Harsch IA. Reduced frequency of peripheral dendritic cells in type 2 diabetes. Exp Clin Endocrinol Diabetes. (2008) 116:162–6. 10.1055/s-2007-990278 18213547

[140] Blank SE Johnson EC Weeks DK Wysham CH. Circulating dendritic cell number and intracellular TNF-alpha production in women with type 2 diabetes. Acta Diabetol. (2012) 49(Suppl. 1):S25–32. 10.1007/s00592-010-0190-8 20449757

[141] Gilardini Montani MS Granato M Cuomo L Valia S Di Renzo L D'orazi G. High glucose and hyperglycemic sera from type 2 diabetic patients impair DC differentiation by inducing ROS and activating Wnt/β-catenin and p38 MAPK. Biochim Biophys Acta. (2016) 1862:805–13. 10.1016/j.bbadis.2016.01.001 26769359

[142] Swirski FK Libby P Aikawa E Alcaide P Luscinskas FW Weissleder R. Ly-6Chi monocytes dominate hypercholesterolemia-associated monocytosis and give rise to macrophages in atheromata. J Clin Invest. (2007) 117:195–205. 10.1172/JCI29950 17200719 PMC1716211

[143] Gonzalez L Trigatti BL. Macrophage apoptosis and necrotic core development in atherosclerosis: a rapidly advancing field with clinical relevance to imaging and therapy. Can J Cardiol. (2017) 33:303–12. 10.1016/j.cjca.2016.12.010 28232016

[144] Zhang X Huang F Li W Dang J-L Yuan J Wang J. Human gingiva-derived mesenchymal stem cells modulate monocytes/macrophages and alleviate atherosclerosis. Front Immunol. (2018) 9:878. 10.3389/fimmu.2018.00878 29760701 PMC5937358

[145] Ma H Liu G Ding W Wu Y Cai L Zhao Y. Diabetes-induced alteration of F4/80+ macrophages: a study in mice with streptozotocin-induced diabetes for a long term. J Mol Med. (2008) 86:391–400. 10.1007/s00109-008-0304-8 18231763

[146] Sun C Sun L Ma H Peng J Zhen Y Duan K. The phenotype and functional alterations of macrophages in mice with hyperglycemia for long term. J Cell Physiol. (2012) 227:1670–9. 10.1002/jcp.22891 21678423

[147] Kousathana F Georgitsi M Lambadiari V Giamarellos-Bourboulis EJ Dimitriadis G Mouktaroudi M. Defective production of interleukin-1 beta in patients with type 2 diabetes mellitus: Restoration by proper glycemic control. Cytokine. (2017) 90:177–84. 10.1016/j.cyto.2016.11.009 27918955

[148] Khanna S Biswas S Shang Y Collard E Azad A Kauh C. Macrophage dysfunction impairs resolution of inflammation in the wounds of diabetic mice. PLoS ONE. (2010) 5:e9539. 10.1371/journal.pone.0009539 20209061 PMC2832020

[149] Mirza RE Fang MM Weinheimer-Haus EM Ennis WJ Koh TJ. Sustained inflammasome activity in macrophages impairs wound healing in type 2 diabetic humans and mice. Diabetes. (2014) 63:1103–14. 10.2337/db13-0927 24194505 PMC3931398

[150] Mirza RE Fang MM Novak ML Urao N Sui A Ennis WJ. Macrophage PPARgamma and impaired wound healing in type 2 diabetes. J Pathol. (2015) 236:433–44. 10.1002/path.4548 25875529 PMC4509817

[151] Inoue H Shirakawa J Togashi Y Tajima K Okuyama T Kyohara M. Signaling between pancreatic beta cells and macrophages via S100 calcium-binding protein A8 exacerbates beta-cell apoptosis and islet inflammation. J Biol Chem. (2018) 293:5934–46. 10.1074/jbc.M117.809228 29496993 PMC5912464

[152] Westwell-Roper CY Ehses JA Verchere CB. Resident macrophages mediate islet amyloid polypeptide-induced islet IL-1beta production and beta-cell dysfunction. Diabetes. (2014) 63:1698–711. 10.2337/db13-0863 24222351

[153] Ward MG Li G Hao M. Apoptotic beta-cells induce macrophage reprogramming under diabetic conditions. J Biol Chem. (2018) 293:16160–73. 10.1074/jbc.RA118.004565 30213857 PMC6200952

[154] Mayadas TN Cullere X Lowell CA. The multifaceted functions of neutrophils. Annu Rev Pathol. (2014) 9:181–218. 10.1146/annurev-pathol-020712-164023 24050624 PMC4277181

[155] Ridzuan N John CM Sandrasaigaran P Maqbool M Liew LC Lim J. Preliminary study on overproduction of reactive oxygen species by neutrophils in diabetes mellitus. World J Diabetes. (2016) 7:271–8. 10.4239/wjd.v7.i13.271 27433296 PMC4937165

[156] Tessari P Coracina A Kiwanuka E Vedovato M Vettore M Valerio A. Effects of insulin on methionine and homocysteine kinetics in type 2 diabetes with nephropathy. Diabetes. (2005) 54:2968–76. 10.2337/diabetes.54.10.2968 16186400

[157] Wong SL Demers M Martinod K Gallant M Wang Y Goldfine AB. Diabetes primes neutrophils to undergo NETosis, which impairs wound healing. Nat Med. (2015) 21:815–9. 10.1038/nm.3887 26076037 PMC4631120

[158] Joshi MB Baipadithaya G Balakrishnan A Hegde M Vohra M Ahamed R. Elevated homocysteine levels in type 2 diabetes induce constitutive neutrophil extracellular traps. Sci Rep. (2016) 6:36362. 10.1038/srep36362 27811985 PMC5095649

[159] Jain SK Bull R Rains JL Bass PF Levine SN Reddy S. Low levels of hydrogen sulfide in the blood of diabetes patients and streptozotocin-treated rats causes vascular inflammation? Antioxid Redox Signal. (2010) 12:1333–7. 10.1089/ars.2009.2956 20092409 PMC2935346

[160] Shefa U Kim M-S Jeong NY Jung J Antioxidant and cell-signaling functions of hydrogen sulfide in the central nervous system. Oxid Med Cell Longev. (2018) 2018:1873962 10.1155/2018/1873962 29507650 PMC5817206

[161] Yang CT Chen L Chen WL Li N Chen MJ Li X. Hydrogen sulfide primes diabetic wound to close through inhibition of NETosis. Mol Cell Endocrinol. (2019) 480:74–82. 10.1016/j.mce.2018.10.013 30339820

[162] Fadini GP Menegazzo L Rigato M Scattolini V Poncina N Bruttocao A. NETosis delays diabetic wound healing in mice and humans. Diabetes. (2016) 65:1061–71. 10.2337/db15-0863 26740598

[163] Wang L Zhou X Yin Y Mai Y Wang D Zhang X. Hyperglycemia induces neutrophil extracellular traps formation through an nadph oxidase-dependent pathway in diabetic retinopathy. Front Immunol. (2019) 9:3076. 10.3389/fimmu.2018.03076 30671057 PMC6331470

[164] Gómez García A Rivera Rodríguez M Gómez Alonso C Rodríguez Ochoa DY Alvarez Aguilar C. Myeloperoxidase is associated with insulin resistance and inflammation in overweight subjects with first-degree relatives with type 2 diabetes mellitus. Diabetes Metab J. (2015) 39:59–65. 10.4093/dmj.2015.39.1.59 25729714 PMC4342538

[165] Zhang C Yang J Jennings LK. Leukocyte-derived myeloperoxidase amplifies high-glucose-induced endothelial dysfunction through interaction with high-glucose–stimulated, vascular non-leukocyte-derived reactive oxygen species. Diabetes. (2004) 53:2950–9. 10.2337/diabetes.53.11.2950 15504976

[166] Alba-Loureiro TC Munhoz CD Martins JO Cerchiaro GA Scavone C Curi R. Neutrophil function and metabolism in individuals with diabetes mellitus. Braz J Med Biol Res. (2007) 40:1037–44. 10.1590/S0100-879X2006005000143 17665039

[167] Kuwabara WMT Yokota CNF Curi R Alba-Loureiro TC. Obesity and type 2 diabetes mellitus induce lipopolysaccharide tolerance in rat neutrophils. Sci Rep. (2018) 8:17534. 10.1038/s41598-018-35809-2 30510205 PMC6277411

[168] Kraakman MJ Lee MK Al-Sharea A Dragoljevic D Barrett TJ Montenont E. Neutrophil-derived S100 calcium-binding proteins A8/A9 promote reticulated thrombocytosis and atherogenesis in diabetes. J Clin Invest. (2017) 127:2133–47. 10.1172/JCI92450 28504650 PMC5451242

[169] Thom SR Bhopale VM Yu K Huang W Kane MA Margolis DJ. Neutrophil microparticle production and inflammasome activation by hyperglycemia due to cytoskeletal instability. J Biol Chem. (2017) 292:18312–24. 10.1074/jbc.M117.802629 28972154 PMC5672053

[170] Leroyer AS Tedgui A Boulanger CM. Microparticles and type 2 diabetes. Diabetes Metab. (2008) 34 Suppl 1:S27–32. 10.1016/S1262-3636(08)70100-9 18358424

[171] França CN Izar MCDO Amaral JBD Tegani DM Fonseca FAH. Microparticles as potential biomarkers of cardiovascular disease. Arq Bras Cardiol. (2015) 104:169–74. 10.5935/abc.20140210 25626759 PMC4375661

[172] Guillot R Bringuier AF Porokhov B Guillausseau PJ Feldmann G. Increased levels of soluble Fas in serum from diabetic patients with neuropathy. Diabetes Metab. (2001) 27:315–21. 11431596

[173] Margaryan S Witkowicz A Arakelyan A Partyka A Karabon L Manukyan G. sFasL-mediated induction of neutrophil activation in patients with type 2 diabetes mellitus. PLoS ONE. (2018) 13:e0201087. 10.1371/journal.pone.0201087 30024959 PMC6053218

[174] Piatkiewicz P Milek T Bernat-Karpinska M Ohams M Czech A Ciostek P. The dysfunction of NK cells in patients with type 2 diabetes and colon cancer. Arch Immunol Ther Exp. (2013) 61:245–53. 10.1007/s00005-013-0222-5 23456207

[175] Piatkiewicz P Bernat-Karpinska M Milek T Rabijewski M Rosiak E. NK cell count and glucotransporter 4 (GLUT4) expression in subjects with type 2 diabetes and colon cancer. Diabetol Metab Syndr. (2016) 8:38. 10.1186/s13098-016-0152-6 27303448 PMC4906701

[176] Berrou J Fougeray S Venot M Chardiny V Gautier J-F Dulphy N. Natural killer cell function, an important target for infection and tumor protection, is impaired in type 2 diabetes. PLoS ONE. (2013) 8:e62418. 10.1371/journal.pone.0062418 23638076 PMC3636194

[177] Peraldi MN Berrou J Dulphy N Seidowsky A Haas P Boissel N. Oxidative stress mediates a reduced expression of the activating receptor NKG2D in NK cells from end-stage renal disease patients. J Immunol. (2009) 182:1696–705. 10.4049/jimmunol.182.3.1696 19155520

[178] Coquet JM Chakravarti S Kyparissoudis K Mcnab FW Pitt LA Mckenzie BS. Diverse cytokine production by NKT cell subsets and identification of an IL-17-producing CD4-NK1.1- NKT cell population. Proc Natl Acad Sci USA. (2008) 105:11287–92. 10.1073/pnas.0801631105 18685112 PMC2516267

[179] Phoksawat W Jumnainsong A Leelayuwat N Leelayuwat C. Aberrant NKG2D expression with IL-17 production of CD4+ T subsets in patients with type 2 diabetes. Immunobiology. (2017) 222:944–51. 10.1016/j.imbio.2016.05.001 27168217

[180] Phoksawat W Jumnainsong A Leelayuwat N Leelayuwat C. IL-17 production by NKG2D-expressing CD56+ T cells in type 2 diabetes. Mol Immunol. (2018) 106:22–8. 10.1016/j.molimm.2018.12.008 30576948

[181] Lv X Gao Y Dong T Yang L. Role of natural killer T (NKT) cells in type II diabetes-induced vascular injuries. Med Sci Monit. (2018) 24:8322–32. 10.12659/MSM.912446 30451213 PMC6256848

[182] Artis D Spits H. The biology of innate lymphoid cells. Nature. (2015) 517:293–301. 10.1038/nature14189 25592534

[183] Spits H Artis D Colonna M Diefenbach A Di Santo JP Eberl G. Innate lymphoid cells-a proposal for uniform nomenclature. Nat Rev Immunol. (2013) 13:145–9. 10.1038/nri3365 23348417

[184] Liu F Wang H Feng W Ye X Sun X Jiang C. Type 1 innate lymphoid cells are associated with type 2 diabetes. Diabetes Metab. (2019) 45:341–6. 10.1016/j.diabet.2018.08.005 30189343

[185] Wang H Shen L Sun X Liu F Feng W Jiang C. Adipose group 1 innate lymphoid cells promote adipose tissue fibrosis and diabetes in obesity. Nat Commun. (2019) 10:3254. 10.1038/s41467-019-11270-1 31332184 PMC6646407

[186] Liu C Qin L Ding J Zhou L Gao C Zhang T. Group 2 innate lymphoid cells participate in renal fibrosis in diabetic kidney disease partly via TGF-β1 signal pathway. J Diabetes Res. (2019) 2019:8512028. 10.1155/2019/8512028 31355294 PMC6636594

[187] Galle-Treger L Sankaranarayanan I Hurrell BP Howard E Lo R Maazi H. Costimulation of type-2 innate lymphoid cells by GITR promotes effector function and ameliorates type 2 diabetes. Nat Commun. (2019) 10:713. 10.1038/s41467-019-08449-x 30755607 PMC6372786

[188] Dalmas E Lehmann FM Dror E Wueest S Thienel C Borsigova M. Interleukin-33-activated islet-resident innate lymphoid cells promote insulin secretion through myeloid cell retinoic acid production. Immunity. (2017) 47:928–42. 10.1016/j.immuni.2017.10.015 29166590

[189] Danze PM Tarjoman A Rousseaux J Fossati P Dautrevaux M. Evidence for an increased glycation of IgG in diabetic patients. Clin Chim Acta. (1987) 166:143–53. 10.1016/0009-8981(87)90416-5 3621595

[190] Kennedy DM Skillen AW Self CH. Glycation of monoclonal antibodies impairs their ability to bind antigen. Clin Exp Immunol. (1994) 98:245–51. 10.1111/j.1365-2249.1994.tb06133.x 7955529 PMC1534406

[191] Lapolla A Tonani R Fedele D Garbeglio M Senesi A Seraglia R. Non-enzymatic glycation of IgG: an *in vivo* study. Horm Metab Res. (2002) 34:260–4. 10.1055/s-2002-32140 12063640

[192] Vrdoljak A Trescec A Benko B Hecimovic D Simic M. *In vitro* glycation of human immunoglobulin G. Clin Chim Acta. (2004) 345:105–11. 10.1016/j.cccn.2004.03.026 15193984

[193] Goodarzi MT Ghahramany S Mirmomeni MH Glycation of human IgG induces structural alterations leading to changes in its interaction with anti-IgG. Iran J Immunol. (2005) 2:36–42.

[194] Shcheglova T Makker S Tramontano A. Reactive immunization suppresses advanced glycation and mitigates diabetic nephropathy. J Am Soc Nephrol. (2009) 20:1012–9. 10.1681/ASN.2008050555 19389854 PMC2678040

[195] Pozzilli P Gale E Visallil N Baroni M Crovari P Frighi V. The immune response to influenza vaccination in diabetic patients. Diabetologia. (1986) 29:850–4. 10.1007/BF00870139 3569690

[196] Diepersloot R Bouter K Beyer W Hoekstra J Masurel N. Humoral immune response and delayed type hypersensitivity to influenza vaccine in patients with diabetes mellitus. Diabetologia. (1987) 30:397–401. 10.1007/BF00292541 3678660

[197] Diepersloot R Bouter K Van Beek R Lucas C Masurel N Erkelens D. Cytotoxic T-cell response to influenza A subunit vaccine in patients with type 1 diabetes mellitus. Neth J Med. (1989) 35:68–75. 2789342

[198] Sheridan PA Paich HA Handy J Karlsson EA Schultz-Cherry S Hudgens M. The antibody response to influenza vaccination is not impaired in type 2 diabetics. Vaccine. (2015) 33:3306–13. 10.1016/j.vaccine.2015.05.043 26044491 PMC4593058

[199] Farnsworth CW Shehatou CT Maynard R Nishitani K Kates SL Zuscik MJ. A humoral immune defect distinguishes the response to Staphylococcus aureus infections in mice with obesity and type 2 diabetes from that in mice with type 1 diabetes. Infect Immun. (2015) 83:2264–74. 10.1128/IAI.03074-14 25802056 PMC4432732

[200] Farnsworth CW Schott EM Benvie A Kates SL Schwarz EM Gill SR. Exacerbated Staphylococcus aureus foot infections in obese/diabetic mice are associated with impaired germinal center reactions, Ig class switching, and humoral immunity. J Immunol. (2018) 201:560–72. 10.4049/jimmunol.1800253 29858265 PMC6039240

[201] Mathews CE Brown EL Martinez PJ Bagaria U Nahm MH Burton RL Impaired function of antibodies to pneumococcal surface protein A but not to capsular polysaccharide in Mexican American adults with type 2 diabetes mellitus. Clin Vaccine Immunol. (2012) 19:1360–9. 10.1128/CVI.00268-12 22761295 PMC3428395

[202] Kalantar F Dabbaghmanesh MH Martinuzzi E Moghadami M Amirghofran Z. Islet amyloid polypeptide is not a target antigen for CD8+ T-cells in type 2 diabetes. Iran J Immunol. (2014) 11:1–12. 24632583 10.22034/iji.2014.16760

[203] Kumar NP Sridhar R Nair D Banurekha VV Nutman TB Babu S. Type 2 diabetes mellitus is associated with altered CD8(+) T and natural killer cell function in pulmonary tuberculosis. Immunology. (2015) 144:677–86. 10.1111/imm.12421 25363329 PMC4368174

[204] Moura J Rodrigues J Gonçalves M Amaral C Lima M Carvalho E. Impaired T-cell differentiation in diabetic foot ulceration. Cell Mol Immunol. (2017) 14:758–69. 10.1038/cmi.2015.116 26996067 PMC5596240

[205] Richard C Wadowski M Goruk S Cameron L Sharma AM Field CJ. Individuals with obesity and type 2 diabetes have additional immune dysfunction compared with obese individuals who are metabolically healthy. BMJ Open Diabetes Res Care. (2017) 5:e000379. 10.1136/bmjdrc-2016-000379 28761653 PMC5530252

[206] Martinez PJ Mathews C Actor JK Hwang SA Brown EL De Santiago HK. Impaired CD4+ and T-helper 17 cell memory response to *Streptococcus pneumoniae* is associated with elevated glucose and percent glycated hemoglobin A1c in Mexican Americans with type 2 diabetes mellitus. Transl Res. (2014) 163:53–63. 10.1016/j.trsl.2013.07.005 23927943 PMC3954646

[207] Marques JM Rial A Munoz N Pellay FX Van Maele L Leger H Protection against *Streptococcus pneumoniae* serotype 1 acute infection shows a signature of Th17- and IFN-gamma-mediated immunity. Immunobiology. (2012) 217:420–9. 10.1016/j.imbio.2011.10.012 22204818

[208] Leung OM Li J Li X Chan VW Yang KY Ku M. Regulatory T cells promote apelin-mediated sprouting angiogenesis in type 2 diabetes. Cell Rep. (2018) 24:1610–26. 10.1016/j.celrep.2018.07.019 30089270

[209] Perl A Gergely P Nagy G Koncz A Banki K. Mitochondrial hyperpolarization: a checkpoint of T-cell life, death and autoimmunity. Trends immunol. (2004) 25:360–7. 10.1016/j.it.2004.05.001 15207503 PMC4034110

[210] Cho YM Park KS Lee HK. Genetic factors related to mitochondrial function and risk of diabetes mellitus. Diabetes Res Clin Pract. (2007) 77:S172–7. 10.1016/j.diabres.2007.01.052 17451836

[211] Patti M-E Corvera S. The role of mitochondria in the pathogenesis of type 2 diabetes. Endocr Rev. (2010) 31:364–95. 10.1210/er.2009-0027 20156986 PMC3365846

[212] Khan S Raghuram GV Bhargava A Pathak N Chandra DH Jain SK. Role and clinical significance of lymphocyte mitochondrial dysfunction in type 2 diabetes mellitus. Transl Res. (2011) 158:344–59. 10.1016/j.trsl.2011.08.007 22061042

[213] Kumar S Ramachandran R Mete U Mittal T Dutta P Kumar V. Acute pyelonephritis in diabetes mellitus: Single center experience. Indian J Nephrol. (2014) 24:367–71. 10.4103/0971-4065.135347 25484530 PMC4244716

[214] Malazy OT Shariat M Heshmat R Majlesi F Alimohammadian M Tabari NK. Vulvovaginal candidiasis and its related factors in diabetic women. Taiwan J Obstet Gynecol. (2007) 46:399–404. 10.1016/S1028-4559(08)60010-8 18182346

[215] Nitzan O Elias M Chazan B Saliba W. Urinary tract infections in patients with type 2 diabetes mellitus: review of prevalence, diagnosis, and management. Diabetes Metab Syndr Obes. (2015) 8:129–36. 10.2147/DMSO.S51792 25759592 PMC4346284

[216] Javid A Zlotnikov N Pětrošová H Tang TT Zhang Y Bansal AK. Hyperglycemia impairs neutrophil-mediated bacterial clearance in mice infected with the lyme disease pathogen. PLoS ONE. (2016) 11:e0158019. 10.1371/journal.pone.0158019 27340827 PMC4920391

[217] Yano H Kinoshita M Fujino K Nakashima M Yamamoto Y Miyazaki H. Insulin treatment directly restores neutrophil phagocytosis and bactericidal activity in diabetic mice and thereby improves surgical site *Staphylococcus aureus* infection. Infect Immun. (2012) 80:4409–16. 10.1128/IAI.00787-12 23027538 PMC3497398

[218] Lin JC Siu LK Fung CP Tsou HH Wang JJ Chen CT. Impaired phagocytosis of capsular serotypes K1 or K2 *Klebsiella pneumoniae* in type 2 diabetes mellitus patients with poor glycemic control. J Clin Endocrinol Metab. (2006) 91:3084–7. 10.1210/jc.2005-2749 16720670

[219] Chanchamroen S Kewcharoenwong C Susaengrat W Ato M Lertmemongkolchai G. Human polymorphonuclear neutrophil responses to *Burkholderia pseudomallei* in healthy and diabetic subjects. Infect Immun. (2009) 77:456–63. 10.1128/IAI.00503-08 18955471 PMC2612264

[220] Riyapa D Buddhisa S Korbsrisate S Cuccui J Wren BW Stevens MP. Neutrophil extracellular traps exhibit antibacterial activity against *Burkholderia pseudomallei* and are influenced by bacterial and host factors. Infect Immun. (2012) 80:3921–9. 10.1128/IAI.00806-12 22927051 PMC3486034

[221] Easton A Haque A Chu K Lukaszewski R Bancroft GJ. A critical role for neutrophils in resistance to experimental infection with *Burkholderia pseudomallei*. J Infect Dis. (2007) 195:99–107. 10.1086/509810 17152013

[222] Lopez-Lopez N Martinez AGR Garcia-Hernandez MH Hernandez-Pando R Castañeda-Delgado JE Lugo-Villarino G. Type-2 diabetes alters the basal phenotype of human macrophages and diminishes their capacity to respond, internalise, and control *Mycobacterium tuberculosis*. Mem Inst Oswaldo Cruz. (2018) 113:170326. 10.1590/0074-02760170326 29513874 PMC5851047

[223] Martinez N Ketheesan N West K Vallerskog T Kornfeld H. Impaired recognition of *Mycobacterium tuberculosis* by alveolar macrophages from diabetic mice. J Infect Dis. (2016) 214:1629–37. 10.1093/infdis/jiw436 27630197 PMC5144731

[224] Tripathi D Radhakrishnan RK Sivangala Thandi R Paidipally P Devalraju KP Neela VSK. IL-22 produced by type 3 innate lymphoid cells (ILC3s) reduces the mortality of type 2 diabetes mellitus (T2DM) mice infected with Mycobacterium tuberculosis. PLoS Pathog. (2019) 15:e1008140. 10.1371/journal.ppat.1008140 31809521 PMC6919622

[225] Graves DT Kayal RA. Diabetic complications and dysregulated innate immunity. Front Biosci. (2008) 13:1227–39. 10.2741/2757 17981625 PMC3130196

[226] Thimmappaiah Jagadeesh A Prakash PY Karthik Rao N Ramya V. Culture characterization of the skin microbiome in type 2 diabetes mellitus: a focus on the role of innate immunity. Diabetes Res Clin Pract. (2017) 134:1–7. 10.1016/j.diabres.2017.09.007 28951341

[227] Alitalo A Meri T Rämö L. Jokiranta TS Heikkilä T Seppälä IJ. Complement evasion by *Borrelia burgdorferi*: serum-resistant strains promote C3b inactivation. Infect Immun. (2001) 69:3685–91. 10.1128/IAI.69.6.3685-3691.2001 11349031 PMC98369

[228] Garnett JP Baker EH Naik S Lindsay JA Knight GM Gill S. Metformin reduces airway glucose permeability and hyperglycaemia-induced *Staphylococcus aureus* load independently of effects on blood glucose. Thorax. (2013) 68:835–45. 10.1136/thoraxjnl-2012-203178 23709760 PMC3756442

[229] Hodgson KA Govan BL Walduck AK Ketheesan N Morris JL. Impaired early cytokine responses at the site of infection in a murine model of type 2 diabetes and melioidosis comorbidity. Infect Immun. (2013) 81:470–7. 10.1128/IAI.00930-12 23208607 PMC3553796

[230] Buddhisa S Rinchai D Ato M Bancroft GJ Lertmemongkolchai G. Programmed death ligand 1 on *Burkholderia pseudomallei*-infected human polymorphonuclear neutrophils impairs T cell functions. J Immunol. (2015) 194:4413–21. 10.4049/jimmunol.1402417 25801435

[231] Kronsteiner B Chaichana P Sumonwiriya M Jenjaroen K Chowdhury FR Chumseng S. Diabetes alters immune response patterns to acute melioidosis in humans. Eur J Immunol. (2019) 49:1092–106. 10.1002/eji.201848037 31032897 PMC6618312

[232] Nielsen TB Pantapalangkoor P Yan J Luna BM Dekitani K Bruhn K. Diabetes exacerbates infection via hyperinflammation by signaling through TLR4 and RAGE. MBio. (2017) 8:e00818–e00817. 10.1128/mBio.00818-17 28830942 PMC5565964

[233] Asante-Poku A Asare P Baddoo NA Forson A Klevor P Otchere ID. TB-diabetes co-morbidity in Ghana: the importance of Mycobacterium africanum infection. PLoS ONE. (2019) 14:e0211822. 10.1371/journal.pone.0211822 30730937 PMC6366779

[234] Martinez N Kornfeld H. Diabetes and immunity to tuberculosis. Eur J Immunol. (2014) 44:617–26. 10.1002/eji.201344301 24448841 PMC4213860

[235] Restrepo BI. Diabetes and tuberculosis. Microbiol Spectr. (2016) 4:1–11. 10.1128/microbiolspec.TNMI7-0023-2016 28084206 PMC5240796

[236] Kumar NP Sridhar R Banurekha VV Jawahar MS Fay MP Nutman TB. Type 2 diabetes mellitus coincident with pulmonary tuberculosis is associated with heightened systemic type 1, type 17, and other proinflammatory cytokines. Ann Am Thorac Soc. (2013) 10:441–9. 10.1513/AnnalsATS.201305-112OC 23987505 PMC3960913

[237] Tan KS Lee KO Low KC Gamage AM Liu Y Tan GY. Glutathione deficiency in type 2 diabetes impairs cytokine responses and control of intracellular bacteria. J Clin Invest. (2012) 122:2289–300. 10.1172/JCI57817 22546856 PMC3366396

[238] Chellan G Shivaprakash S Ramaiyar SK Varma AK Varma N Sukumaran MT. Spectrum and prevalence of fungi infecting deep tissues of lower-limb wounds in patients with type 2 diabetes. J Clin Microbiol. (2010) 48:2097–102. 10.1128/JCM.02035-09 20410345 PMC2884499

[239] Woldemariam HK Geleta DA Tulu KD Aber NA Legese MH Fenta GM. Common uropathogens and their antibiotic susceptibility pattern among diabetic patients. BMC Infect Dis. (2019) 19:43. 10.1186/s12879-018-3669-5 30630427 PMC6327582

[240] Li J-Z Li J-Y Wu T-F Xu J-H Huang C-Z Cheng D. *Helicobacter pylori* infection is associated with type 2 diabetes, not type 1 diabetes: an updated meta-analysis. Gastroenterol Res Pract. (2017) 2017:5715403. 10.1155/2017/5715403 28883831 PMC5572635

[241] Cui M Fang Q Zheng J Shu Z Chen Y Fan Y. Kaposi's sarcoma associated herpesvirus seropositivity is associated with type 2 diabetes mellitus: a case-control study in Xinjiang, China. Int J Infect Dis. (2019) 80:73–9. 10.1016/j.ijid.2019.01.003 30639407

[242] Yang J Feng Y Yuan M Yuan S Fu H Wu B. Plasma glucose levels and diabetes are independent predictors for mortality and morbidity in patients with SARS. Diabet Med. (2006) 23:623–8. 10.1111/j.1464-5491.2006.01861.x 16759303

[243] Alraddadi BM Watson JT Almarashi A Abedi GR Turkistani A Sadran M. Risk factors for primary middle east respiratory syndrome coronavirus illness in humans, Saudi Arabia, 2014. Emerg Infect Dis. (2016) 22:49–55. 10.3201/eid2201.151340 26692185 PMC4696714

[244] Ramana BV Babu KVS Chaudhury A Prevalence of hepatitis c virus infection in type 2 diabetic patients at a tertiary care hospital. J Diabetes Res. (2016) 2:23–5. 10.1155/2013/539361

[245] Farshadpour F Taherkhani R Ravanbod MR Eghbali SS. Prevalence and genotype distribution of hepatitis C virus infection among patients with type 2 diabetes mellitus. Med Princ Pract. (2018) 27:308–16. 10.1159/000488985 29621783 PMC6170927

[246] Villar LM Geloneze B Vasques ACJ Pires MLE Miguel JC Da Silva EF. Prevalence of hepatitis B and hepatitis C among diabetes mellitus type 2 individuals. PLoS ONE. (2019) 14:e0211193. 10.1371/journal.pone.0211193 30817756 PMC6394929

[247] Kumar M Roe K Nerurkar PV Namekar M Orillo B Verma S. Impaired virus clearance, compromised immune response and increased mortality in type 2 diabetic mice infected with West Nile virus. PLoS ONE. (2012) 7:e44682. 10.1371/journal.pone.0044682 22953001 PMC3432127

[248] Juttada U Smina TP Kumpatla S Viswanathan V. Seroprevalence and risk factors associated with HBV and HCV infection among subjects with type 2 diabetes from South India. Diabetes Res Clin Pract. (2019) 153:133–7. 10.1016/j.diabres.2019.06.003 31189089

[249] Short KR Richard M Verhagen JH Van Riel D Schrauwen EJ Van Den Brand JM. One health multiple challenges: the inter-species transmission of influenza A virus. One Health. (2015) 1:1–13. 10.1016/j.onehlt.2015.03.001 26309905 PMC4542011

[250] Hulme KD Gallo LA Short KR. Influenza virus and glycemic variability in diabetes: a killer combination? Front Microbiol. (2017) 8:861. 10.3389/fmicb.2017.00861 28588558 PMC5438975

[251] Allard R Leclerc P Tremblay C Tannenbaum T-N. Diabetes and the severity of pandemic influenza A (H1N1) infection. Diabetes care. (2010) 33:1491–3. 10.2337/dc09-2215 20587722 PMC2890346

[252] Wilking H Buda S Lippe EVD Altmann D Krause G Eckmanns T. Mortality of 2009 pandemic influenza A (H1N1) in Germany. Euro Surveill. (2010) 15:19741. 10.2807/ese.15.49.19741-en 21163179

[253] Roberts BW Cech I. Association of type 2 diabetes mellitus and seroprevalence for cytomegalovirus. South Med J. (2005) 98:686–92. 10.1097/01.SMJ.0000163310.12516.2D 16108236

[254] Sun Y Pei W Wu Y Yang Y. An Association of herpes simplex virus type 1 infection with type 2 diabetes. Diabetes Care. (2005) 28:435–6. 10.2337/diacare.28.2.435 15677810

[255] Ke CC Lai HC Lin CH Hung CJ Chen DY Sheu WH. Increased risk of Herpes Zoster in diabetic patients comorbid with coronary artery disease and microvascular disorders: a population-based study in Taiwan. PLoS ONE. (2016) 11:e0146750. 10.1371/journal.pone.0146750 26751202 PMC4709044

[256] Fung TS Liu DX. Human coronavirus: host-pathogen interaction. Annu Rev Microbiol. (2019) 73:529–57. 10.1146/annurev-micro-020518-115759 31226023

[257] Shirato K Kawase M Matsuyama S. Middle East respiratory syndrome coronavirus infection mediated by the transmembrane serine protease TMPRSS2. J Virol. (2013) 87:12552–61. 10.1128/JVI.01890-13 24027332 PMC3838146

[258] Coutard B Valle C De Lamballerie X Canard B Seidah NG Decroly E. The spike glycoprotein of the new coronavirus 2019-nCoV contains a furin-like cleavage site absent in CoV of the same clade. Antiviral Res. (2020) 176:104742. 10.1016/j.antiviral.2020.104742 32057769 PMC7114094

[259] Hoffmann M Kleine-Weber H Schroeder S Krüger N Herrler T Erichsen S. SARS-CoV-2 cell entry depends on ACE2 and TMPRSS2 and is blocked by a clinically proven protease inhibitor. Cell. (2020) 181:271–80.e8. 10.1016/j.cell.2020.02.052 32142651 PMC7102627

[260] Du L Zhao G Kou Z Ma C Sun S Poon VKM Identification of a receptor-binding domain in the s protein of the novel human coronavirus middle east respiratory syndrome coronavirus as an essential target for vaccine development. J Virol. (2013) 87:9939–42. 10.1128/JVI.01048-13 23824801 PMC3754113

[261] Mönkemüller K Fry L Rickes S Covid-19, Coronavirus, SARS-CoV-2 and the small bowel. Rev Esp Enferm Dig. (2020) 112:383–8. 10.17235/reed.2020.7137/2020 32343593

[262] Abassi ZA Skorecki K Heyman SN Kinaneh S Armaly Z. Covid-19 infection and mortality: a physiologist's perspective enlightening clinical features and plausible interventional strategies. Am J Physiol Lung Cell Mol Physiol. (2020) 318:L1020–2. 10.1152/ajplung.00097.2020 32207983 PMC7200872

[263] Sungnak W Huang N Bécavin C Berg M Network H SARS-CoV-2 entry genes are most highly expressed in nasal goblet and ciliated cells within human airways. Nat Med. (2020) 26:681–7. 10.1038/s41591-020-0868-6 32327758 PMC8637938

[264] Wan Y Shang J Graham R Baric RS Li F. Receptor recognition by the novel coronavirus from wuhan: an analysis based on decade-long structural studies of SARS coronavirus. J Virol. (2020) 94:e00127–e00120. 10.1128/JVI.00127-20 31996437 PMC7081895

[265] Andersen KG Rambaut A Lipkin WI Holmes EC Garry RF. The proximal origin of SARS-CoV-2. Nat Med. (2020) 26:450–2. 10.1038/s41591-020-0820-9 32284615 PMC7095063

[266] Ji H-L Zhao R Matalon S Matthay MA. Elevated plasmin(ogen) as a common risk factor for COVID-19 susceptibility. Physiol Rev. (2020) 100:1065–75. 10.1152/physrev.00013.2020 32216698 PMC7191627

[267] Suk K Kim S Kim Y-H Kim K-A Chang I Yagita H. IFN-γ/TNF-α synergism as the final effector in autoimmune diabetes: a key role for STAT1/IFN regulatory factor-1 pathway in pancreatic β cell death. J Immunol. (2001) 166:4481–9. 10.4049/jimmunol.166.7.4481 11254704

[268] Hill MA Mantzoros C Sowers JR. Commentary: COVID-19 in patients with diabetes. Metabolism. (2020) 107:154217. 10.1016/j.metabol.2020.154217 32220611 PMC7102643

[269] Wang F Yang Y Dong K Yan Y Zhang S Ren H Clinical characteristics of 28 patients with diabetes and covid-19 in wuhan, china. Endocr Pract. (2020). 10.4158/EP-2020-0108 PMC7414317 32357072

[270] Muniyappa R Gubbi S. COVID-19 pandemic, coronaviruses, and diabetes mellitus. Am J Physiol Endocrinol Metab. (2020) 318:E736–41. 10.1152/ajpendo.00124.2020 32228322 PMC7191633

[271] Singh AK Gupta R Ghosh A Misra A. Diabetes in COVID-19: Prevalence, pathophysiology, prognosis and practical considerations. Diabetes Metab Syndr. (2020) 14:303–10. 10.1016/j.dsx.2020.04.004 32298981 PMC7195120

[272] Guo W Li M Dong Y Zhou H Zhang Z Tian C Diabetes is a risk factor for the progression and prognosis of COVID-19. Diabetes Metab Res Rev. (2020). 10.1002/dmrr.3319. [Epub ahead of print]. PMC7228407 32233013

[273] Tay MZ Poh CM Rénia L Macary PA Ng LFP. The trinity of COVID-19: immunity, inflammation and intervention. Nat Rev Immunol. (2020) 20:363–74. 10.1038/s41577-020-0311-8 32346093 PMC7187672

[274] Diao B Wang C Tan Y Chen X Liu Y Ning L. Reduction and functional exhaustion of t cells in patients with coronavirus disease 2019 (COVID-19). Front Immunol. (2020) 11:827. 10.3389/fimmu.2020.00827 32425950 PMC7205903

[275] Wang F Nie J Wang H Zhao Q Xiong Y Deng L Characteristics of peripheral lymphocyte subset alteration in COVID-19 pneumonia. J Infect Dis. (2020) 221:1762–9. 10.1093/infdis/jiaa150 32227123 PMC7184346

[276] Danquah I Bedu-Addo G Mockenhaupt FP. Type 2 diabetes mellitus and increased risk for malaria infection. Emerg Infect Dis. (2010) 16:1601–4. 10.3201/eid1610.100399 20875289 PMC3294394

[277] Li Y-X Xin H Zhang X-Y Wei C-Y Duan Y-H Wang H-F. *Toxoplasma gondii* infection in diabetes mellitus patients in china: seroprevalence, risk factors, and case-control studies. Biomed Res Int. (2018) 2018:4723739. 10.1155/2018/4723739 30662909 PMC6312584

[278] Htun NSN Odermatt P Paboriboune P Sayasone S Vongsakid M Phimolsarn-Nusith V. Association between helminth infections and diabetes mellitus in adults from the Lao People's Democratic Republic: a cross-sectional study. Infect Dis Poverty. (2018) 7:105. 10.1186/s40249-018-0488-2 30396368 PMC6219195

[279] Mendonca SC Goncalves-Pires Mdo R Rodrigues RM Ferreira AJr Costa-Cruz JM. Is there an association between positive Strongyloides stercoralis serology and diabetes mellitus? Acta Trop. (2006) 99:102–5. 10.1016/j.actatropica.2006.06.006 16872576

[280] Alemu G Jemal A Zerdo Z. Intestinal parasitosis and associated factors among diabetic patients attending Arba Minch Hospital, Southern Ethiopia. BMC Res Notes. (2018) 11:689. 10.1186/s13104-018-3791-x 30285833 PMC6167831

[281] Mohtashamipour M Ghaffari Hoseini SG Pestehchian N Yousefi H Fallah E Hazratian T Intestinal parasitic infections in patients with diabetes mellitus: a case-control study. J Anal Res Clin Med. (2015) 3:157–63. 10.15171/jarcm.2015.025

[282] Akinbo FO Olujobi SO Omoregie R Egbe CJB Medicine G Intestinal parasitic infections among diabetes mellitus patients. Biomarkers Genomic Med. (2013) 5:44–7. 10.1016/j.gmbhs.2013.05.003

[283] Machado ER Matos NO Rezende SM Carlos D Silva TC Rodrigues L. Host-parasite interactions in individuals with type 1 and 2 diabetes result in higher frequency of ascaris lumbricoides and giardia lamblia in type 2 diabetic individuals. J Diabetes Res. (2018) 2018:4238435. 10.1155/2018/4238435 29541642 PMC5818974

[284] Omaña-Molina M Sanchez-Rocha R Hernandez-Martinez D Romero Grijalva M Salinas-Lara C Rodriguez-Sosa M. Type 2 diabetes mellitus BALB/c mice are more susceptible to granulomatous amoebic encephalitis: immunohistochemical study. Exp Parasitol. (2017) 183:150–9. 10.1016/j.exppara.2017.09.001 28917708

[285] Al Mubarak S Robert AA Baskaradoss JK Al-Zoman K Al Sohail A Alsuwyed A. The prevalence of oral Candida infections in periodontitis patients with type 2 diabetes mellitus. J Infect Public Health. (2013) 6:296–301. 10.1016/j.jiph.2012.12.007 23806705

